# Plasmacytoid dendritic cells at the forefront of anti-cancer immunity: rewiring strategies for tumor microenvironment remodeling

**DOI:** 10.1186/s13046-024-03121-9

**Published:** 2024-07-17

**Authors:** Matilde Monti, Giorgia Ferrari, Luisa Gazzurelli, Mattia Bugatti, Fabio Facchetti, William Vermi

**Affiliations:** 1 Department of Molecular and Translational Medicine, Section of Pathology, University of Brescia, P.Le Spedali Civili 1, 25123 Brescia, Italy; 2 Department of Pathology and Immunology, Washington University School of Medicine, Saint Louis, MO USA

**Keywords:** Plasmacytoid dendritic cells, Cancer, Immune surveillance, Tumor microenvironment, Type I Interferon, Cytotoxic function, Immunometabolism, TLR7/9 agonists, STING agonists, Clinical trials

## Abstract

Plasmacytoid dendritic cells (pDCs) are multifaceted immune cells executing various innate immunological functions. Their first line of defence consists in type I interferons (I-IFN) production upon nucleic acids sensing through endosomal Toll-like receptor (TLR) 7- and 9-dependent signalling pathways. Type I IFNs are a class of proinflammatory cytokines that have context-dependent functions on cancer immunosurveillance and immunoediting. In the last few years, different studies have reported that pDCs are also able to sense cytosolic DNA through cGAS–STING (stimulator of interferon genes) pathway eliciting a potent I-IFN production independently of TLR7/9. Human pDCs are also endowed with direct effector functions via the upregulation of TRAIL and production of granzyme B, the latter modulated by cytokines abundant in cancer tissues. pDCs have been detected in a wide variety of human malignant neoplasms, including virus-associated cancers, recruited by chemotactic stimuli. Although the role of pDCs in cancer immune surveillance is still uncompletely understood, their spontaneous activation has been rarely documented; moreover, their presence in the tumor microenvironment (TME) has been associated with a tolerogenic phenotype induced by immunosuppressive cytokines or oncometabolites. Currently tested treatment options can lead to pDCs activation and disruption of the immunosuppressive TME, providing a relevant clinical benefit. On the contrary, the antibody–drug conjugates targeting BDCA-2 on immunosuppressive tumor-associated pDCs (TA-pDCs) could be proposed as novel immunomodulatory therapies to achieve disease control in patients with advance stage hematologic malignancies or solid tumors. This Review integrate recent evidence on the biology of pDCs and their pharmacological modulation, suggesting their relevant role at the forefront of cancer immunity.

## Background

Plasmacytoid dendritic cells (pDCs) are bone marrow-derived circulating innate immune cells, identified as CD123^+^ (IL3R), CD303^+^ (BDCA-2, also known as C-type lectin CLEC4C), CD304^+^ (BDCA-4) cells, and negative for lineage markers and CD11c [1].

pDCs home to lymphoid organs and inflamed peripheral tissues by chemotactic stimuli [2], where they execute relevant immune effector and regulatory functions upon nucleic acids sensing through different surface and cytosolic receptors. pDCs have been detected in a wide variety of human neoplasms, including carcinomas [e.g. uroendothelial bladder cancer (UBC); colon-rectal adenocarcinomas (CAD); lung squamous cell carcinoma (LSCC); head neck squamous cell carcinoma (HNSCC)], cutaneous melanoma (CM), lymphomas, and virus-associated cancers (e.g., cervical cancers, oral cancers, nasopharyngeal carcinoma, Kaposi’s sarcoma) [3, 4]. In the last decade, studies have proposed a relevant role for pDC also in cancer immunity. Their expansion and activation in the tumor tissue by recently developed compounds might promote a fine-tuning remodelling of the tumor microenviroment (TME). The present review will summarize recent advances on the role of pDCs in cancer by focusing on their intrinsic antitumor activity and functional states in cancer tissues. Based on these findings, this review will propose combinatorial strategies to overcome cellular and molecular immune escape mechanisms targeting pDCs biology.

### The multifaceted biology of pDC in cancer

pDCs are multifaceted immune cells, executing innate immunological functions [5]. Their first line of defense against viral infections and cancer consists of type I interferons (I-IFNs) production upon sensing nucleic acids through endosomal Toll-like receptor (TLR) 7- and 9-dependent signaling pathways [6, 7]. Besides TLRs, human pDCs express different nucleic acid sensors, such as C-type lectin receptors (CLRs), RIG-I-like receptors (RLRs), NOD-like receptors (NLRs), and cyclic guanosine monophosphate-adenosine monophosphate synthase (cGAS) [8, 9].

Moreover, pDCs are producers of type III interferons (IFN-λ or IL-28/IL-29) and have been identified among the restricted cell subsets also expressing the IFN-λ functional receptor (IFNλR) [10]. As previously reviewed [11], III-IFNs can serve as an autocrine signal to potentiate the anti-viral and antitumor activities of pDCs by triggering IFN-α and IFN-λ production [12, 13], and it also influences the activation status of pDCs by upregulating CD80 and CD86. In addition to IFNs production, pDCs could exert direct cytotoxic functions or potentiate Natural Killer (NK) cells effector functions. By TLR7/9 engagement, activated pDCs could exert killing capacities via the upregulation of TNF-related apoptosis inducing ligand (TRAIL) [14, 15]. Moreover, granzyme B (GrB) is constitutively expressed in human pDCs, but its production and release are further induced by the cytokines abundant in cancer tissues [15–17].

Tumor-associated pDCs (TA-pDCs; i.e. into the tumor milieu or tumor-draining lymph nodes) are recruited at the tumor site by chemokines (e.g. CXCL12 and CCL20) released by cancer cells or by the TME. The presence of pDCs in the TME has been associated with different outcomes suggesting their dual functional/regulatory state in cancer immunity. Indeed, they can exhibit either a tolerogenic or immunogenic phenotype, depending on the immunological context. Moreover, pDCs may interact with tumor-associated viruses [e.g. Human Papilloma Virus (HPV), Epstein-Barr Virus (EBV), Human herpes virus 8 (HHV8)] that persist in tumor tissues and may contribute to tumor immunity [4, 18–20]. However, no direct pDC-virus interaction in cancers has been reported as yet. Further studies are necessary to understand the crosstalk between oncogenic viruses and pDCs and whether the tumor-infiltrating pDCs in HPV/EBV/HHV8 positive tumors are immunocompetent. Although the role of pDCs in cancer immunoediting/immune surveillance is still uncompletely understood, a large set of studies have documented that tumor-infiltrating pDCs often exhibit a non-activated and tolerogenic state [21–23]. The non-activated tolerogenic TA-pDCs state could be induced by immunosuppressive cytokines or oncometabolites. Tolerogenic pDCs are characterized by low I-IFNs production and co-stimulatory molecule expression and promote regulatory T cell (Treg) induction together with increased expression of immunomodulatory molecules (e.g. IDO). Accordingly, a high density of TA-pDCs have been frequently associated with a poor clinical outcome. On the other hand, pDCs are endowed with the ability to induce maturation of conventional DC1 (cDC1), can present tumor antigens and prime tumor-specific cytotoxic CD8^+^ T cells [24–26]; however, whether and how human pDCs cross-prime CD8^+^ T cells in vivo has been controversial for a long time. Based upon a recent report, cross-presenting pDCs were unable to prime CD8^+^ T cells efficiently, but require conventional DCs (cDCs) to achieve CD8^+^ T cell cross-priming in vivo [27].

Novel immunotherapeutic approaches aim to disrupt the immunosuppressive TME by reprogramming the tolerogenic phenotype of TA-pDCs and other immune cells, towards an immunogenic phenotype boosting innate and adaptive tumor-specific immune responses.

### News in pDC affiliation

#### Decoding pDC ontogeny

**Fig. 1:**
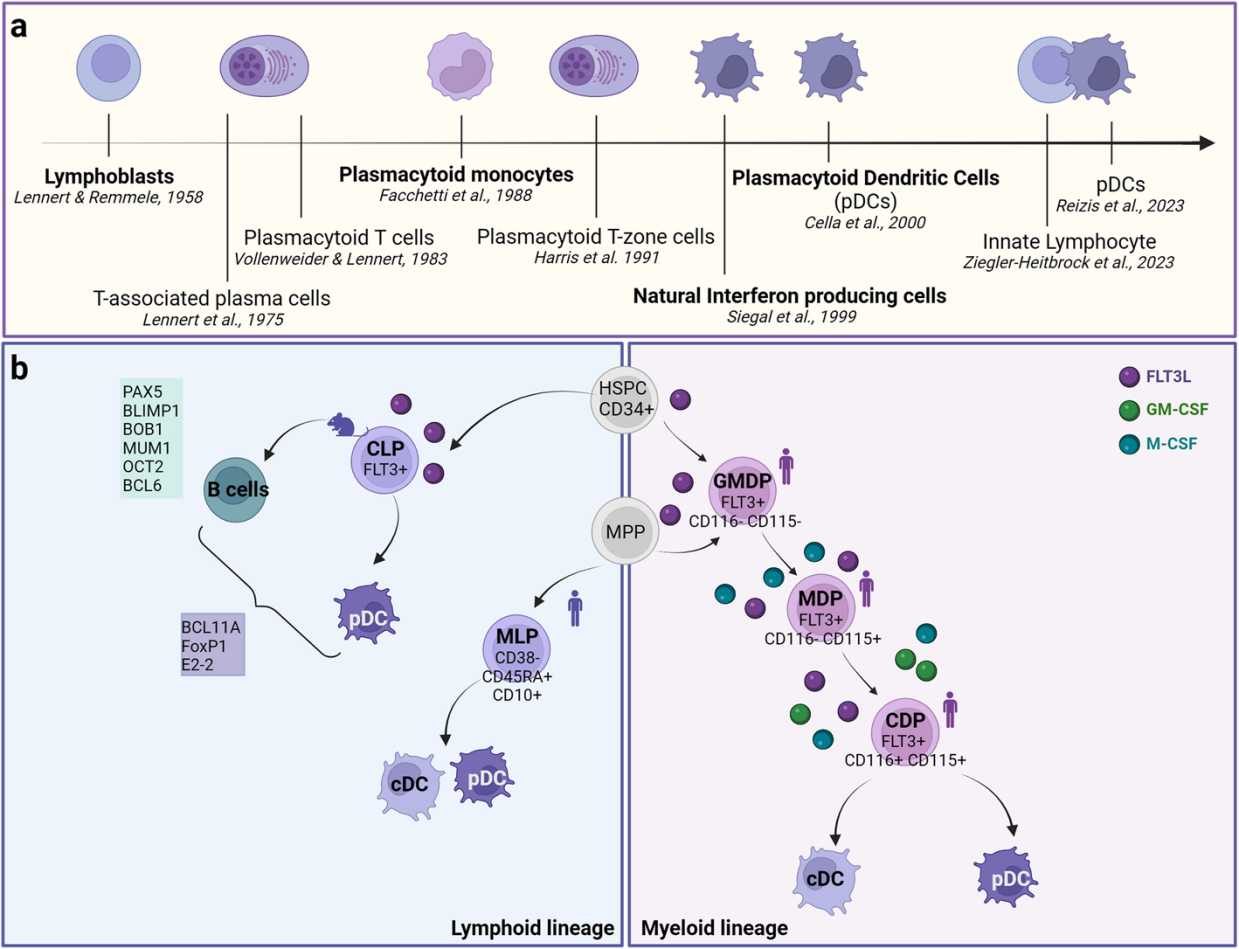
Discovery of plasmacytoid dendritic cells, decoding their ontogeny and differentiation. **a** The timeline clarifies the different nomenclature and origin that have been attributed to human pDCs from their first identification to the present. **b** pDCs share features with both myeloid and lymphoid lineage opening a debate regarding their ontogeny. Two main developmental cascades are proposed to generate fully differentiated pDCs from lymphoid or myeloid progenitors. These findings were obtained from in vitro experiments by using hematopoietic stem progenitor cells (HSPC) from human bone marrow (indicated with human icons) or by using mouse models (indicated with mouse icons). Human pDCs express some B-lineage transcription factors such as E2-2, BCL11A and FOXP1, but lacks expression of most classical transcription factors for B-cells, including PAX5, BLIMP1, BOB1, MUM1/IRF4, OCT2, and BCL6. MPP: multipotent progenitor; CLP: common lymphoid progenitor; MLP: multipotent lymphoid progenitor; GMDP: granulocyte monocyte and dendritic cell progenitor; MDP: monocyte and dendritic cell progenitor; CDP: common dendritic cell progenitor. Created with *BioRender.com*

The dispute over pDC ontogeny and affiliation started from their first identification by Karl Lennert as cells with plasma cell-like morphology located in lymphoid tissues described as “lymphoblasts” [28] (Fig. 1A). Over the decades, hematopathologists referred to these cells as “T-associated plasma cells” [29], “plasmacytoid T cells” [30] and “plasmacytoid T-zone cells” [31]. Subsequently, these cells were characterized in morphology and phenotype and referred as to plasmacytoid monocytes [32, 33] (Fig. 1A). It was then reported that peripheral blood plasmacytoid monocytes can differentiate into dendritic cells under the influence of stimuli, such as viruses, IL-3, and CD40L [34–37]. Through isolation and in vitro studies, pDCs were identified as Natural Interferon Producing Cells [38, 39] (Fig. 1A).

Although the name of this subset can be immediately traced back to classical dendritic cells, the origin of the pDC is still under debate within the scientific community [40–42] and it was supported by the pDC phenotype sharing concomitant features with both myeloid and lymphoid cells [32, 33, 38, 43]. Moreover, plasmacytoid and classic DCs exhibit distinct features in terms of morphology, phenotype, and functions [5, 39, 44–46], raising doubts over whether they belong to the same cell lineage.

Recently, Ziegler-Heitbrock and colleagues proposed reclassifying pDCs as innate lymphocytes, based on developmental trajectory shared with B cells and the scant classic DC functional properties (i.e. antigen presentation and migration via the lymphatics), or restoring their original name, *type I-Interferon producing cells* as the defining functional feature of these cells [40] (Fig. 1A).

It has been reported that pDCs can express lymphoid transcripts including the pre-T cell antigen receptor alpha chain (pT-α), Spi-B, γ5, Pax5, and TdT [47–49], suggesting the lymphoid origin of pDC [50]. Moreover, human pDCs and their neoplastic counterparts are also marked by some B-lineage transcription factors such as BCL11A and FOXP1 [51]. However, the similarities between pDCs and early B cells in terms of molecular features might rely on their expression of E2-2 and E2A/HEB transcription factors, which are known to regulate common target genes [52]. More importantly, human pDCs lack expression of most classic transcription factors for B cells including PAX5, BLIMP1, BOB1, MUM1/IRF4, OCT2, and BCL6 [51]. Thus, these evidences are insufficient to prove the common origin of B cells and pDCs [42]. An early study identified a mouse M-CSFR/CD115^−^ IL-7Rα^−^ FLT3^+^ CDP with the highest expression level of homologous E protein transcription factors (E2-2, also known as Tcf4) and superior potential to generate pDC compared to canonical Lin^−^ cKIT^int/lo^ FLT3^+^ M-CSFR/CD115^+^ CDP, under the same conditions [53], postulating a possible dual origin of pDCs. Despite the fact that it is now collectively accepted that pDCs can develop from both myeloid and lymphoid precursors [54–57] (Fig. 1B), this issue confuses and divides the scientific community regarding pDC classification [40–42]. The entire process of pDC development takes place in the bone marrow (BM) from hematopoietic stem progenitor cells (HSPCs) and is orchestrated by specific cytokine signals. In common with cDCs, pDC development is strongly dependent on fms-like tyrosine kinase-3 ligand (Flt3L) [55, 57, 58] and transcriptional cofactor Trim33 (also known as TIF1-γ) [59]. Flt3L KO mice have reduced levels of pDCs and cDCs [55], and the expression of Flt3L receptor (Flt3/CD135) by BM precursor is essential to produce both murine and human DCs [55, 60]. Moreover, Trim33 deletion in vivo caused rapid loss of pDCs and cDCs affecting the earliest stages of differentiation, but it also abolished mature B cells. In particular, Trim33-deficient Flt3^+^ progenitors failed to induce the DC differentiation program establishing Trim33 as a key transcriptional regulator of Flt3L-driven pDCs and cDCs development [59]. The pivotal role of Flt3L driving pDC development from BM progenitors, but its failure to induce lymphoid cell commitment, was one of the main reasons claimed to justify the close developmental affinity of pDC to cDC lineage [32]. Besides the dependence on FLT3L cytokine, cDC and pDC share the expression of transcription factors, thus supporting a common regulatory network [42, 52, 55, 58]. Notably, pDC development was observed in mice depleted of lymphoid precursor through estrogen therapy [61]. Moreover, the common cytokine receptor γ-chain or the transcription factor JAK3, which are required for the development of lymphocytes, are not required for pDC development [32, 50, 55], further confirming the interrelation of pDCs and cDCs in the developmental process. Finally, pDCs and cDCs share a restricted common dendritic cell progenitor (CDP) through myeloid lineage, with the ability to respond to myeloid cytokines (MCSF, GMCSF) [62]. An in vitro culture system has been used to demonstrate human DC sequential origin from increasingly restricted progenitors [63]. CDP has been proposed as the unique progenitor restricted to DC lineage in humans originated from human monocyte-dendritic progenitor (hMDP), which in turn originates from a human granulocyte-monocyte-DC progenitor (hGMDP) [63]. Subsequently, Helft et al. showed that a large proportion of multipotent lymphoid early progenitors (MLPs) in humans were more efficient in generating CD141^+^ cDCs than common myeloid progenitors (CMPs), and MLPs were also the only progenitors able to generate CD303^+^ pDCs in vitro and in vivo [64]. Recently, clonal lineage tracing of mouse pDCs has confirmed the shared origin of conventional and plasmacytoid DCs [65], strongly in contrast with previous data identifying mouse Ly6D^hi^CD2^hi^ lymphoid progenitors as unique progenitors for pDCs [66]. Pathogenic drivers in neoplastic entities, such as blastic plasmacytoid dendritic cell neoplasm (BPDCN), “Mature PDC Proliferation” (MPDCP), and acute myeloid leukemia (AML)-PDC, are shared by blasts, pDCs, monocytes and cDCs, suggesting a common differentiation trajectory [67, 68] and that a significant proportion of pDC neoplastic proliferation might originate from a myeloid precursor.

In summary, the ontogeny of pDCs remains controversial and a dual myeloid/lymphoid origin of pDCs should be considered in order to justify the pDC heterogeneity and plasticity observed under different conditions. ScRNA-seq studies provided helpful information in the area of pDC ontogeny [61, 66]; however, this intricate issue requires additional studies to be thoroughly understood.

#### Human pDC heterogeneity

Increasing reports have demonstrated the potential heterogeneity of pDC cell populations devoted to specific immune functions [69–76]. However, the mechanisms behind the different pDC functions have not yet been fully elucidated and it is unclear whether the environmental cues, rather than specific genetic programs, contribute to pDC diversification [77–79].

**Fig. 2:**
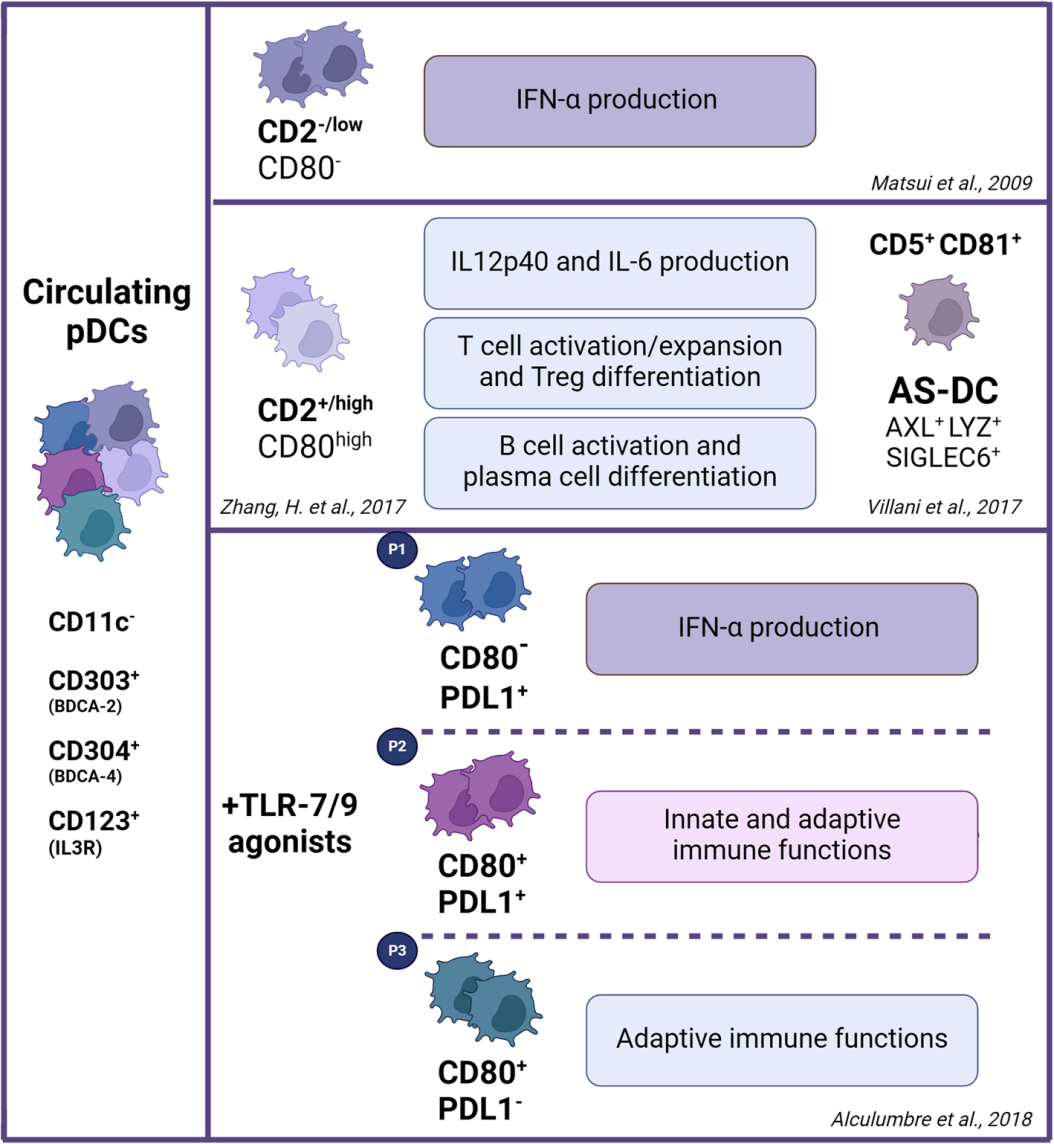
The human circulating pDC subsets. Human pDCs are defined as Lin^−^ CD11c^−^ CD123/IL3R^+^ BDCA-2/CD303^+^ BDCA-4/CD304^+^ cells and designated to IFN-α production. Upon stimulation with TLR7/9 ligands, three human pDC subpopulations were identified as PD-L1^+^CD80^−^ pDCs (P1) specialized in I-IFN production, PD-L1^−^CD80^+^ pDCs (P3) specialized in adaptive immune functions, and PD-L1^+^CD80^+^ pDCs (P2) showing both innate and adaptive immune functions. In unstimulated/basal conditions, no pDCs subsets have been detected. The CD2^+^ pDCs subset most likely corresponds to a new DC cluster, named AS-DC, that is identified as AXL^+^LYZ^+^SIGLEC6^+^ DCs exerting regulatory functions, such as inducing T cells activation and expansion. The expression of AXL and LYZ was also previously observed on the CD5^+^CD81^+^ subset among CD2^+^ pDCs. Created with *BioRender.com*

**Fig. 3:**
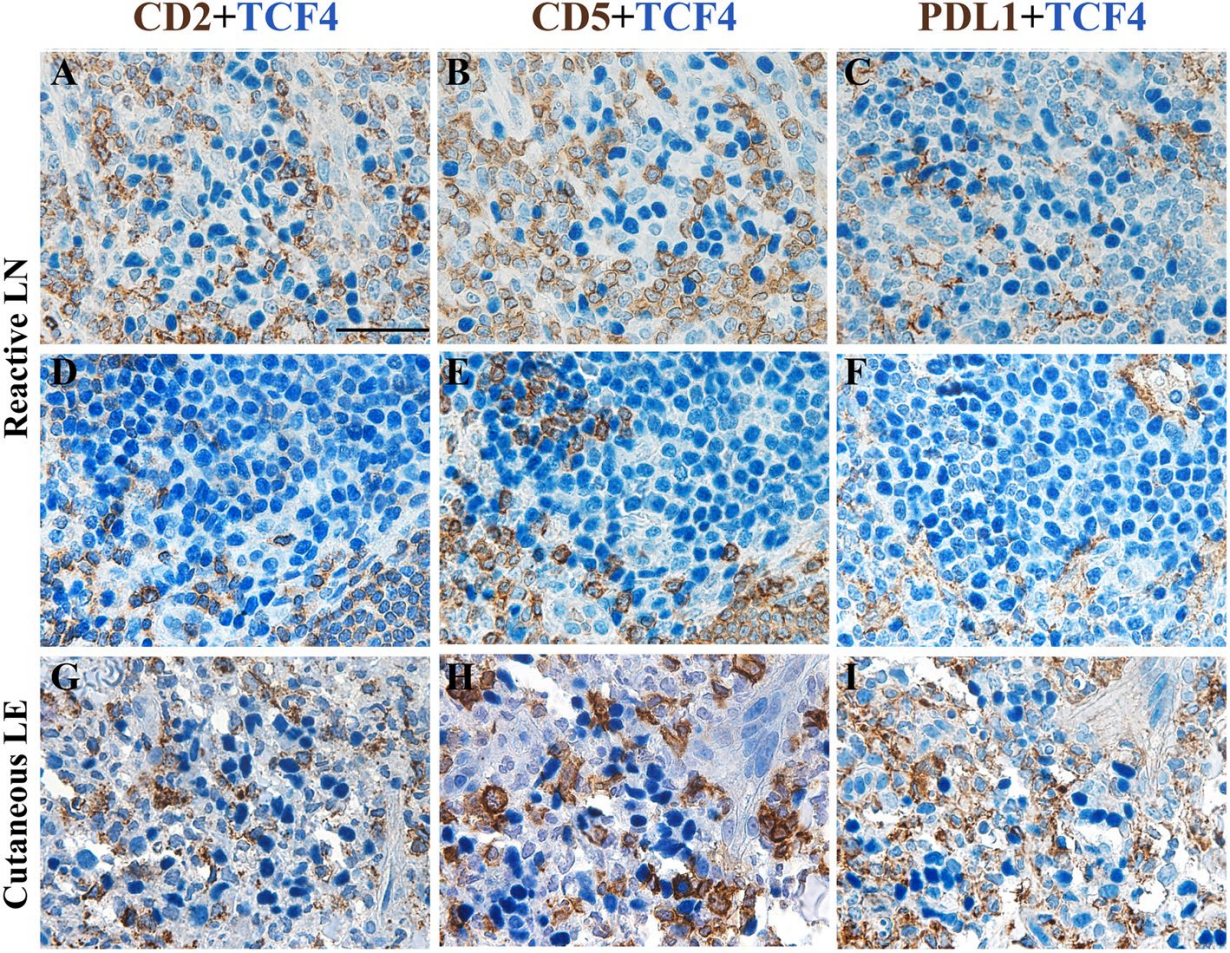
Tissue pDCs are mostly negative for CD2, CD5, and PD-L1. Sections are from reactive lymph nodes (LN) (**a-f**; representative images from 2 out of 5 cases analyzed) and cutaneous lupus erythematosus (LE) (**g-i**; representative images of 1 out of 3 cases analyzed) and stained with TCF4/E2.2, CD2, CD5, and PD-L1, as labeled. Magnification 400x; scale bar 50 µm

Originally, two pDC subsets were identified in human blood and tonsils based on the expression level of CD2 surface antigen: a rare CD2^high^ pDC subset as compared to the major fraction of CD2^low^ pDCs [69, 80] (Fig. 2). CD2^high^ and CD2^low^ pDC subsets showed differentiated ability to initiate T cell immune responses associated with distinct transcriptional profiles. Moreover, upon activation, CD2^high^ pDCs showed higher expression of costimulatory molecule CD80 and higher production of IL-12p40 compared to CD2^low^ pDCs, accompanied by an increased capacity to trigger naive T cell expansion. Of note, CD2^high^ pDCs unexpectedly overexpressed the lysozyme (LYZ), a typical myeloid marker, and the IFN-regulated surface tyrosine kinase receptor AXL. More recently, a further characterization of human pDCs in bone marrow, cord blood, and tonsils enabled the division of CD2^high^ pDCs into CD5^+^CD81^+^ and CD5^−^CD81^−^ subsets both expressing classical pDC surface markers and pDC-defining transcription factors E2-2/TCF4 and SPIB [70, 80]. However, the CD2^high^CD5^+^CD81^+^ pDCs significantly diverged in their transcriptional profile, including a notable overexpression of LYZ and AXL receptor, and functional studies revealed that CD2^high^CD5^+^CD81^+^ pDCs showed a mature phenotype (i.e. costimulatory molecule and HLA-DR expression) and a weak ability to produce IFN-α, consistent with low expression of the Interferon Regulatory Factor 7 (IRF7) [70] (Fig. 2). Specifically, CD5^+^CD81^+^ pDCs were able to produce large quantities of proinflammatory cytokines, such as IL-12p40 and IL-6, promoting T cell proliferation and inducing their differentiation into Treg [70]. A further functional feature of CD5^+^CD81^+^ pDCs was their ability to activate B cells and induce plasma cell differentiation and antibody production dependent on pDC–B cell contact through CD70-CD27 interaction [70]. It should be noted that tissue pDCs are mostly negative for CD2 and CD5 [33, 51], as revealed by immunostaining on reactive lymph nodes and cutaneous lupus erythematosus (Fig. 3), suggesting that phenotypic diversification in different microenvironments [81] or different developmental phases might occur. This finding could be also explained by the absent/low expression of surface CCR7 on CD2^+^CD5^+^Axl^+^ DCs [82]. Based on single-cell transcriptomic profiling and functional studies, human blood DCs and monocytes were reclassified into six different clusters, among which cluster 6 (DC6) mapped to pDCs [71]. Of relevance, a new DC subset characterized by AXL and SIGLEC6 expression was identified and designated as cluster 5 (DC5) AS-DCs. The AS-DCs were found in blood and lymphoid organs, but are absent in peripheral tissues, such as skin [81]. Based on gene expression analysis, AS-DCs exhibited a spectrum of states ranging from a pDC-like signature (e.g., IL3RA, IGJ, NRP1, MZB1) to a cDC2-like signature (e.g., IFI30, ITGAX, LY86, GLIPR2, FGR, LYZ, ENTPD1) suggesting a relationship between these subsets. By flow cytometry analysis, AS-DCs were found within both cDC (CD123^low^CD11c^+^) and pDC (CD123^+^CD11c^−^) gates [71]. Although CD123^+^CD11c^−^ AS-DCs expressed pDC markers (e.g., CD123 and CD303), they are functionally distinct from pDCs. As compared to IFN-α producing pDCs, AS-DCs are more potent activators of T cells. Interestingly, the previously described CD2^high^ pDC subset [69, 70] most likely correspond to AS-DCs in terms of phenotype (i.e. CD2, AXL, CX3CR1, LYZ and CD86 expression) and regulatory functions (i.e. naïve T cells activation and proliferation) [71] (Fig. 2). Therefore, some functions previously attributed to pDCs could be due to contaminating AS-DCs and functional studies should be performed by excluding Axl^+^ DCs. Finally, the identity and developmental trajectory of AS-DCs [72] is still a matter of debate and further studies will be required to determine whether Axl^+^ DCs constitute a distinct DC subset [71] or are circulating DC progenitors [83].

In recent years, pDC heterogeneity has been revealed as a wide range of diversification upon stimulation [73–76]. Three divergent human pDC subpopulations were identified upon activation in response to a single innate stimulus, based on programmed death-ligand 1 (PD-L1) and CD80 expression [73] (Fig. 2). These subpopulations showed distinct functional features associated with specific transcriptional signatures. The P1 pDCs (PD-L1^+^CD80^−^) displayed plasmacytoid morphology and were specialized in I-IFN production, the P3 pDCs (PD-L1^−^CD80^+^) exhibited dendritic morphology and performed regulatory immune functions (i.e. T cell activation and T helper 2 (Th2) differentiation), and the P2 pDCs (PD-L1^+^CD80^+^) represented an intermediate subset [73]. Although their proportions varied among donors, P3-pDCs appeared to be less abundant than P1-pDCs and P2-pDCs. Moreover, the P1-pDC and P3-pDC phenotypes were regulated by different kinetics, being the first and the last to appear after stimuli, respectively. However, the activated pDC populations displayed a phenotypic stability, with or without a second viral challenge, thus indicating that P1, P2, and P3 pDCs are stable cellular entities in diverse functional states. P1-pDCs were prevalent in IFN-α-mediated autoimmune diseases (e.g., lupus and psoriasis) [73], whereas P3-pDCs were increased in melanoma and negatively impacted clinical outcomes [84]. Therefore, CD80 and PD-L1 surface markers can be used as biomarkers to track pDCs with innate or adaptive functions in pathological conditions. However, it should be noted that in reactive lymph nodes and cutaneous lupus erythematosus most pDCs lack PD-L1 (Fig. 3) as well as E2-2/TCF4^+^ BPDCN neoplastic cells [85].

Subsequently, a single-cell analysis using a droplet-based microfluidic platform revealed that TLR-induced IFN-α production by pDCs closely depends on stochastic gene regulation and is amplified by I-IFN paracrine signals in the microenvironment [74]. Only a limited pool (1–3%) of pDCs responded promptly to TLR7/8 and TLR9 agonists (i.e. first responders) and were defined by high surface levels of PD-L1 and TRAIL. The early responders triggered the surrounding pDCs to generate robust I-IFN responses (i.e. second responders) by inducing specific factors [75]. pDC diversification and functional specialization was independent of preexisting heterogeneity within steady-state pDCs, but intercellular crosstalk might be involved in the pDC activation process [73, 74]. Furthermore, distinct and stable pDC populations have been identified in REX3 transgenic mice based on CXCL10 expression, a downstream effector of I-IFN signaling, following TLR7 stimulation. The CXCL10^+^ and CXCL10^−^ pDC populations were defined by different transcriptional programs; however, no current markers are able to define CXCL10 expression potential [76].

Finally, a recent study integrating single-cell RNA sequencing and proteomic data revealed the existence of specialized clusters among human pDCs at baseline or upon activation with influenza virus [86]. Of relevance, this study clearly demonstrated that a single cluster of pDCs is accountable for the majority of induced cytokines, including I-IFNs and III-IFNs [86]; however, no surface markers could discriminate these pDC clusters.

The heterogeneity within human pDCs reflects their competence in orchestrating diverse immune functions, such as effectors of viral responses and drivers of inflammation, activation of adaptive immune cells, and immune tolerance. Nevertheless, these investigations were mainly limited to the healthy circulating pDCs and additional data are needed on human tissue pDCs as well as in the setting of autoimmune diseases and cancers.

### pDCs and cancer immune surveillance

#### pDCs migration toward tumor sites

In homeostatic conditions, fully differentiated pDCs migrate from the bone marrow towards primary and secondary lymphoid tissues via high endothelial venules (HEV) [87]. In inflammatory diseases and cancers, human pDCs can be also found in peripheral non-lymphoid tissues [2]. Fully differentiated pDCs isolated from human blood highly express a set of chemokine receptors, including CCR7, CCR2, CCR5, CXCR4, and CXCR3, weakly express CCR1, CCR4, and CXCR2, whereas CCR6, CXCR1, and CXCR5 are absent on pDC subset [88]. The pDCs migrate in response to the stromal cell–derived factor 1 (SDF-1/CXCL12), a homeostatic chemokine expressed by HEV [88, 89], but also detected in melanoma [43, 90], oral squamous cell carcinoma (OSCC) [91, 92], ovarian cancer [93], breast cancer and metastatic lymph nodes [94, 95], underlining its role in pDC recruitment to tumor sites [87, 88, 93]. The functional role of CXCR3 in chemotactic response by pDCs is still controversial, and it has been previously reviewed [2, 96]. Of relevance, CXCR3 ligands released during inflammation (e.g., Mig/CXCL9 and ITAC/CXCL11) synergize with the constitutive chemokine CXCL12 to induce pDC recruitment. The adjacent and simultaneous co-expression of CXCL12, CXCL9 and CXCL11 could regulate the recruitment of pDCs at sites of inflammation [87].

Peripheral blood pDCs in stage I-III melanoma patients upregulated CCR6, a skin homing receptor, and were recruited to primary cutaneous melanoma lesions, in response to CCL20 produced in melanoma microenvironment [97]. The IL-3 growth factor is able to upregulate CCR6 expression on human blood pDCs [98]; however, it is so far undetermined whether IL-3 or other factors produced by cells of the TME are responsible for CCR6 upregulation by melanoma-associated pDCs. On the contrary, CCR6 expression was low on circulating pDCs from metastatic melanoma (MM) patients (stage IV, AJCC) and comparable to control pDCs [98, 99]. The small subset of blood pDCs expressing both CCR6 and CCR10 might originate from lymph nodes and represent a subset in transit to the inflamed epithelia [98]. Furthermore, CCR7 triggers pDC migration towards CCL19 and CCL21, especially upon activation with TLR ligands, contributing to the pDC homing to draining lymph nodes both at steady state and in inflammatory conditions [82]. Our group has also identified chemerin/RARRES2, the natural ligand of Chemerin Receptor 1, as a new chemotactic factor for human pDCs transiting toward lymphoid tissues and inflamed skin (i.e. lupus erythematosus and psoriasis) [100]. Chemerin is usually expressed by keratinocytes, fibroblasts and blood endothelial cells in inflamed skin and by HEV in reactive lymph nodes [2], but bioactive chemerin was also found in ascitic fluids secondary to ovarian carcinomas [101]. Despite many studies suggested a downregulation or loss of chemerin in various tumors compared to the normal tissue counterparts [102], upregulation of chemerin was found in gastric cancer [103, 104], mesothelioma [105], neuroblastoma [106] and cervical neoplastic lesions [107], suggesting that chemerin in the TME might have context-dependent effects on tumorigenesis and tumor progression.

#### Type I interferon production by pDCs is impaired by the tumor microenvironment

Type I IFNs are a class of proinflammatory cytokines (i.e. including 13 genes coding for IFN-α, and individual gene coding for IFN-β, IFN-ω, IFN-ɛ, and IFN-к) that have context-dependent functions on cancer immunosurveillance and immunoediting [108]. Depending on the dose and timing, and the downstream induced signatures, I-IFNs can foster or prevent tumor progression and immune evasion [109]. Specifically, robust, acute, and ultimately resolving I-IFN responses have been shown to participate in anticancer immunosurveillance and mediate prominent anticancer effects, such as direct cytostatic/cytotoxic activity on malignant cells, but also immunostimulatory functions. On the other hand, weak, sub-optimal, and chronic I-IFN signaling supports tumor progression and resistance to therapy by cancer cell-intrinsic effects (i.e. mediating cytoprotective activity, promoting epithelial-to-mesenchymal transition and stemness) and establishment of an immunologically exhausted TME [109]. pDCs are known as professional IFN-α producing cells and generate 10 to 100 times more IFN-α than other cell types upon activation of pattern-recognition receptors (PRRs). Specifically, TLR7 recognizes single-stranded RNA of viral origin and synthetic mimicking ligands [e.g. imidazoquinoline compounds, such as Imiquimod (IMQ) and Resiquimod (R848)]; whereas TLR9 recognizes microbial unmethylated CpG DNA sequences as well as CpG-rich oligodeoxynucleotides (CpG-ODNs). After TLR7/9 engagement in the early endosomes, the IFN-α response is induced through the cascade activation of the myeloid differentiation primary response protein 88 (MyD88) that interacts with the Interferon Regulatory Factor 7 (IRF7) [110, 111], as described elsewhere in detail [112, 113]. In recent years, different studies reported the capability of pDCs to sense cytosolic DNA through the cGAS–STING (stimulator of interferon genes) pathway eliciting potent I-IFNs production independently of TLR7/9 [114–116]. Briefly, cGAS interacts with double-stranded DNA (dsDNA) and induces conformational changes into 2’3’-cyclic GMP-AMP (cGAMP). The second messenger cGAMP then activates STING [117], which recruits the TANK binding kinase-1 (TBK1) that is phosphorylated and, in turn, phosphorylates and induces nuclear translocation of the Interferon Regulatory Factor 3 (IRF3) [118]. It should be noted that the IRF7-dependent I-IFN signaling pathway produces 100 times more cytokine than the IRF3-dependent I-IFN signaling pathway.

**Fig. 4:**
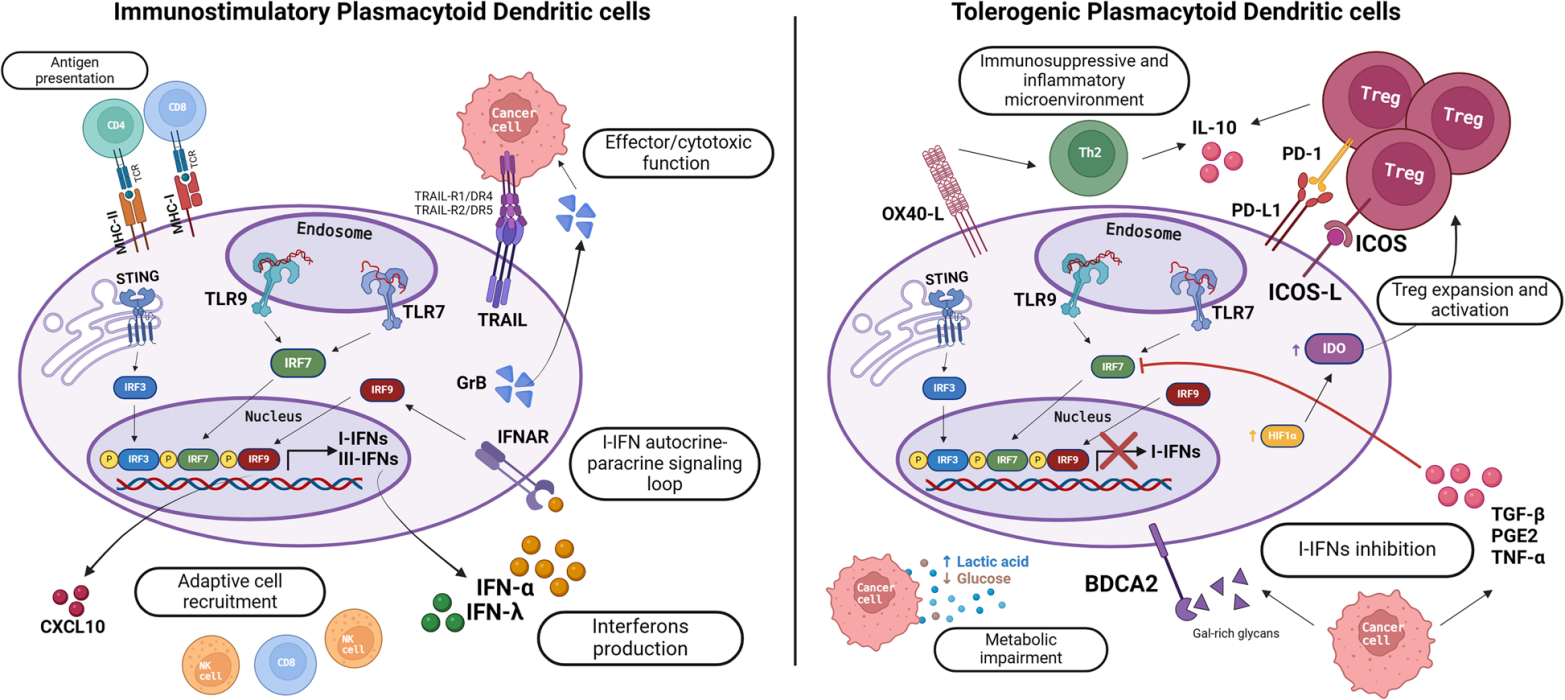
The tolerogenic and immunostimulatory roles of pDCs in the tumor microenvironment. Graphical illustration of the human pDCs polarization in cancer. pDCs can exert anti-tumor and immunostimulatory functions (left panel), whereas tolerogenic pDCs promote the immune evasion of tumor cells (right panel). Tolerogenic pDCs are depicted as functionally impaired (e.g. I-IFN production) cells by tumor-derived cytokines and oncometabolites. Tolerogenic pDCs are further characterized by expression of costimulatory receptors or coinhibitory molecules and generate an immunosuppressive and inflammatory TME (e.g. Treg expansion). On the contrary, properly activated pDCs (e.g. via TLR7/9 agonists or STING agonists) produce type I and type III IFNs and proinflammatory cytokines (e.g. CXCL10), eliciting the recruitment of adaptive immune cells. Human pDCs can exert direct effector functions against tumor cells, as well. The surface molecules and intracellular mechanisms involved in these functions are detailed. Created with *BioRender.com*

TA-pDCs could be functionally impaired by tumor-derived soluble factors released in the TME and then acquire an immunosuppressive profile [21]. Specifically, the pDC proficiency in producing IFN-α can be hijacked by immunosuppressive cytokines, oncometabolites, or ligands to inhibitory receptors expressed on the cell surface, thus supporting cancer progression [22] (Fig. 4). Among the large set of immunosuppressive molecules produced by tumor cells, TGF-β is one of the most studied soluble factors responsible for the functional inhibition of pDCs [119]. TGF-β has been detected in a wide range of cancers, alone [3, 119] or in combination with other immunosuppressive cytokines, such as IL-10 [120, 121], PGE2 [122] and TNF-α [121, 123]. Specifically, the synergy between these cytokines was accountable for the TLR7/9 signaling inhibition, including IRF7 downregulation, impairing the I-IFN production by pDCs [124, 125]. Accordingly, our group proposed a TGF-β-mediated functional impairment of TLR/MyD88-dependent signaling in melanoma-associated pDCs through IRF7 downregulation [126]. Wnt5a is an immunosuppressive molecule identified in the melanoma TME affecting pDC function [127]. As described in the previous section, HMGB1 produced by neoplastic keratinocytes was implicated in the tolerogenic switch of TA-pDCs and decreased the IFN-α secretion upon stimulation with TLR9 agonists [107]. A recent study has shown that tumor-derived pDCs were significantly dampened in IFN-α production upon TLR7/9 activation in HPV-negative HNSCC, but were functionally uncompromised in HPV-positive HNSCC [18]. The immunosuppressive cytokine milieu was rich in IL-10 and TNF-α in HPV-negative but not in HPV-positive HNSCC; the resulting functional impairment of tumor-infiltrating pDCs further supported the immunosuppressive TME by promoting the expansion of Tregs in the tumor tissue [18]. Moreover, virus-like particles have been found to activate tumor-infiltrating pDCs [128]. Although direct evidence is lacking, pDC-tumor cell crosstalk might differ between HPV + and HPV- tumors. Finally, KSHV can infect and activate human pDCs through TLR9 signaling, inducing the upregulation of CD83 and CD86 and IFN-α secretion [129]. However, weak human myxovirus resistance protein 1 (MxA) expression was measured on KS cases, suggesting that I-IFN production by pDCs was suppressed probably in relation to KSHV's ability to evade the immune system [130].

Human pDCs express a large set of activating or inhibitory receptors that regulate the amplitude of the I-IFN response and activation state. The inhibitory receptors include the BDCA-2, the immunoglobulin-like transcript 7 (ILT7, also known as LILRA4 and CD85g), the C-type lectin dendritic cell immunoreceptor (DCIR, also known as CLEC4A), and the natural cytotoxicity receptor NKp44 (also known as NCR2) [131–134]. The regulation of I-IFN secretion by these inhibitory receptors restrain autoimmunity but promote tumor growth. Tumor cells express different ligands that could engage inhibitory receptors expressed on pDCs contributing to pDCs exhaustion and poor antitumor response. The galactose-terminated asialo-oligosaccharides, a glycoprotein component also found on tumor cells, have been identified as natural ligands of BDCA-2 [135]. More recently, galactose-terminated glycans binding to BDCA-2 have been detected [136]. Consistently, the Caroline Aspord’s research group has recently demonstrated that tumor cells harbor a specific glycosylation pattern. Specifically, melanomas were enriched in glycans with galactose residues eliciting an inhibitory impact on pDC functionality (i.e. IFN-α production upon stimulation with R848) [137]. Moreover, the plasma-derived heparin has been identified as specific ligand for BDCA-2 resulting in the inhibition of TLR9-driven IFN-α production in human pDCs. In parallel, an activation-dependent soluble form of BDCA-2 has been identified in human plasma and functions as heparin antagonist leading to enhanced TLR9-driven IFN-α production in pDCs [138]. This very novel observation should be integrated in the emerging oncology chapter on the role of heparin and derived-molecules in the treatment of cancer patients [139]. Furthermore, BDCA-2 has been proposed as a novel target for therapy in autoimmune diseases (i.e. systemic lupus erythematosus) [140, 141] and monoclonal antibodies targeting BDCA-2 could represent a treatment option in patients with hematologic malignancies [140–142]. Moreover, the antibody–drug conjugates targeting BDCA-2 could be developed as immunomodulatory therapy to achieve disease control in solid tumors infiltrated by tolerogenic pDCs [141]. The ligand of ILT7 has been identified as bone marrow stromal cell antigen 2 (BST2, also known as CD317) widely expressed in human tissues to varying degrees and reported in several human cancer cell lines, as well as in a wide range of tumors [143–145]. BST2 expression has been associated with cancer progression and invasiveness [146–148]. The crosslinking of ILT7 and BST2 strongly inhibits the production of IFN-α and proinflammatory cytokines (e.g. TNF-α) by pDCs [143, 149, 150]; however, the role of this interaction in cancer is still unclear. pDCs have also been reported to express the vasoactive intestinal peptide (VIP) receptors VPAC1 and VPAC2, and their crosslinking inhibited IFN-α secretion after CpG stimulation [151].

Finally, cancer cells have a high metabolic activity responsible for nutrients (i.e. glucose) deprivation in the TME. The increased glycolytic rate leads to lactate accumulation and environmental acidification in the tumor milieu [152]. As thoroughly described in the following section of this review, pDC functionality has been linked to glycolysis in order to provide the high energy demand for rapid I-IFN production [153]. Our group has demonstrated that IFN-α production by melanoma-exposed pDCs was hijacked and could derive from their metabolic drift [126] (Fig. 4).

#### pDC effector functions in human cancers: a still unexplored field

In addition to their pivotal role in I-IFN production, pDCs could execute direct cytoytoxic functions (Fig. 4), though their role as antitumor effector is still largely unknown and controversial.

In mouse models of melanoma and breast cancer, activated pDCs can directly kill tumor cells through TRAIL and GrB expression [15, 154] and were also able to recruit and activate NK cells and cross-prime cytotoxic T lymphocytes (CTLs) [155], leading to tumor clearance; however, the direct antitumor effector functions of human pDCs should be explored. After TLR7/9 engagement, activated pDCs could exert killing capacities via the upregulation of cytotoxic molecules, such as TRAIL [14, 156]. TRAIL^+^ pDCs target TRAIL-receptor (TRAIL-R) TRAIL-R1/DR4 or TRAIL-R2/DR5 expressing cells [14, 157]. TRAIL has been detected at high levels in the cytoplasm of unstimulated pDCs, suggesting that stimulation with TLR7 ligands (i.e. ssRNA molecules) induced the re-localization of TRAIL to the cell membrane [158]. Human pDC cell line GEN2.2, stimulated with TLR7/9 agonists, is able to lyse tumor cells in a TRAIL-dependent manner [156]. The exposure of pDCs to ssRNA or dsRNA viruses (e.g. HIV, HCV, Dengue virus) induced IFN-α production as well as TRAIL expression [159–161], sustaining the possible TRAIL-mediated cytotoxic role of pDCs in response to viral infections. Significantly, cytotoxic pDCs could directly neutralize tumor cells. For instance, in patients with basal cell carcinoma, pDCs became competent in killing tumor cells through a cell-to-cell TRAIL-dependent interaction after stimulation with either IMQ, CpG, or IFN-α [14, 162]. At the same time, TA-pDCs could induce local antitumor effector responses through the activation of T cells and NK cells [155, 163], a process mainly mediated by I- and III-IFN production [7, 164]. In a neuroblastoma co-culture system, the TLR9-activated pDCs induced the activation of TRAIL^+^ NK cells that ultimately killed tumor cells [165]. The depletion of either pDCs or IFN-α led to a loss of the TRAIL-mediated tumor cell killing by CD14^+^ monocytes [166].

A second cytolytic molecule that plays a central role in the pDC effector functions is the serine protease granzyme B [15, 167, 168] (Fig. 4). GrB is a pro-apoptotic molecule constitutively expressed in human pDCs [169], but its production and release is further induced by cytokines, such as IL-3, IL-10 and IL-21 [168, 170], abundantly expressed in cancer tissues [17, 171, 172]. Surprisingly, activation of pDCs through TLR7/9 agonists and CD40 ligand negatively regulated GrB expression [17], indicating that IFN-α producing pDCs might not be able to exert cytotoxic function. On the other hand, GrB-expressing pDCs have been involved in the suppression of T cell expansion by inducing T cell apoptosis or by hijacking the T cell proliferation in a GrB-dependent and perforin-independent manner [17, 169, 173]. Specifically, GrB levels produced by pDCs inversely correlated with the proliferation of co-incubated T cells in vitro, whereas T cell proliferation was enhanced by administrating anti-GrB neutralizing antibody or a specific substrate inhibitor [169]. pDC-derived GrB activity might also regulate in vivo T cell responses. Functionally, IL-3-activated pDCs delivered GrB to T cells in a cell-contact dependent manner and degraded the T cell receptor (TCR)-zeta chain, which is a substrate for GrB proteolytic activity [169, 174]. These data suggested that GrB^+^ pDCs in the TME could participate in suppressing the expansion of tumor-specific T cells. In conclusion, GrB-expressing pDCs could have regulatory effects on tumor-specific T cells as well as cytotoxic potential toward virus-infected or transformed cells, implying a contribution to cancer progression or elimination, respectively. However, the expression of GrB in tumor-infiltrating pDCs has been poorly investigated so far and further studies are required to understand whether GrB-secreting pDCs play a role in the immune evasion of cancers.

#### Tolerogenic pDCs in human cancer

pDC antitumor functions can be hijacked by TME that rewires pDCs as tolerogenic cells, thus promoting immune evasion. In the latter case, TA-pDCs instruct an immunosuppressive TME by ligands of costimulatory receptors or coinhibitory molecules expression (i.e. IDO, ICOS-L, PD-L1) [175] and Treg induction [176, 177] (Fig. 4).

The inducible co-stimulator ligand (ICOS-L) is expressed on antigen presenting cells (APCs) and tumor cells and binds to ICOS on activated T cells. The ICOS/ICOS-L interaction generates a pro-tumorigenic response mediated by local Treg expansion and IL-10 production by Tregs and Th2 in the TME [178, 179]. An increased expression of ICOS-L by pDCs has been reported in hepatocellular carcinoma (HCC) [180] and ovarian carcinoma [181], in which the presence of TA-pDCs and ICOS^+^ Tregs was predictive for disease progression [182]. In breast carcinoma, the ICOS-ICOS-L interaction promoted the expansion of Tregs and subsequent IL-10 and TGF-β secretion [183, 184]. A recent study, using a co-culture system of pDCs and cervical/vulvar neoplastic keratinocytes, proposed a role for ICOS-L^+^ pDCs in the local Treg expansion throughout cervical cancer progression [107], a mechanism mediated by the secretion of HMGB1 by neoplastic keratinocytes. The combination of ICOS and cytotoxic T-lymphocyte antigen 4 (CTLA-4) or PD-1/PD-L1 blockade could generate antitumor effects, particularly by preventing the interaction between Tregs and ICOS-L^+^ pDCs [178]. In CM context, pDCs expressed ICOS-L together with tumor necrosis factor ligand superfamily member 4 (TNFSF4; also known as OX40-L), modulating T cell response and Th2 polarization [185, 186]. In contrast, TA-pDCs in HNSCC and tumor-draining lymph nodes (TDLNs) that express high levels of OX40 have been demonstrated to synergize with myeloid DCs to induce a potent tumor antigen-specific CD8^+^ T cell response [187].

The indoleamine 2,3-dioxygenase (IDO) is expressed by tolerogenic pDCs and is responsible for the activation of Tregs. Melanoma-associated human pDCs released IDO promoting tumor escape from immune surveillance [188]. IDO-expressing pDCs were also detected in mouse TDLNs, where they can directly activate mature Tregs through PD-L1 [189]. As documented in HCC [190] and HNSCC [191], hypoxia is an environmental factor that induced IDO upregulation by pDCs and thus their tolerogenic state. Particularly, in HCC the IDO upregulation by pDCs was mediated by HIF-1α/CCL20/STAT1 pathway, promoting tumor tolerance and metastatization [190]. Moreover, by targeting fatty acid-binding protein 5 (FABP5), a cellular chaperone of long-chain fatty acids involved in metabolism of lipids, IDO expression on TA-pDCs was reduced and impaired the generation of FOXP3^+^ Tregs in the TME [192].

Although the angiogenic properties of pDCs have been poorly explored as compared to cDCs, pDCs could exert a tumor-promoting role by inducing angiogenesis [193]. Despite the fact that in vitro pDCs were unable to produce vascular endothelial growth factor (VEGF) [194], TA-pDCs in ovarian carcinoma were shown to produce pro-angiogenic and pro-invasive cytokines, such as TNF-α and CXCL8, particularly after CD40L activation [195]. In addition, IL-1α produced by TA-pDCs was found to be responsible for promoting cell proliferation and angiogenesis in non-small cell lung cancer (NSCLC) [196].

#### The prognostic role of tumor-associated pDCs

**Fig. 5:**
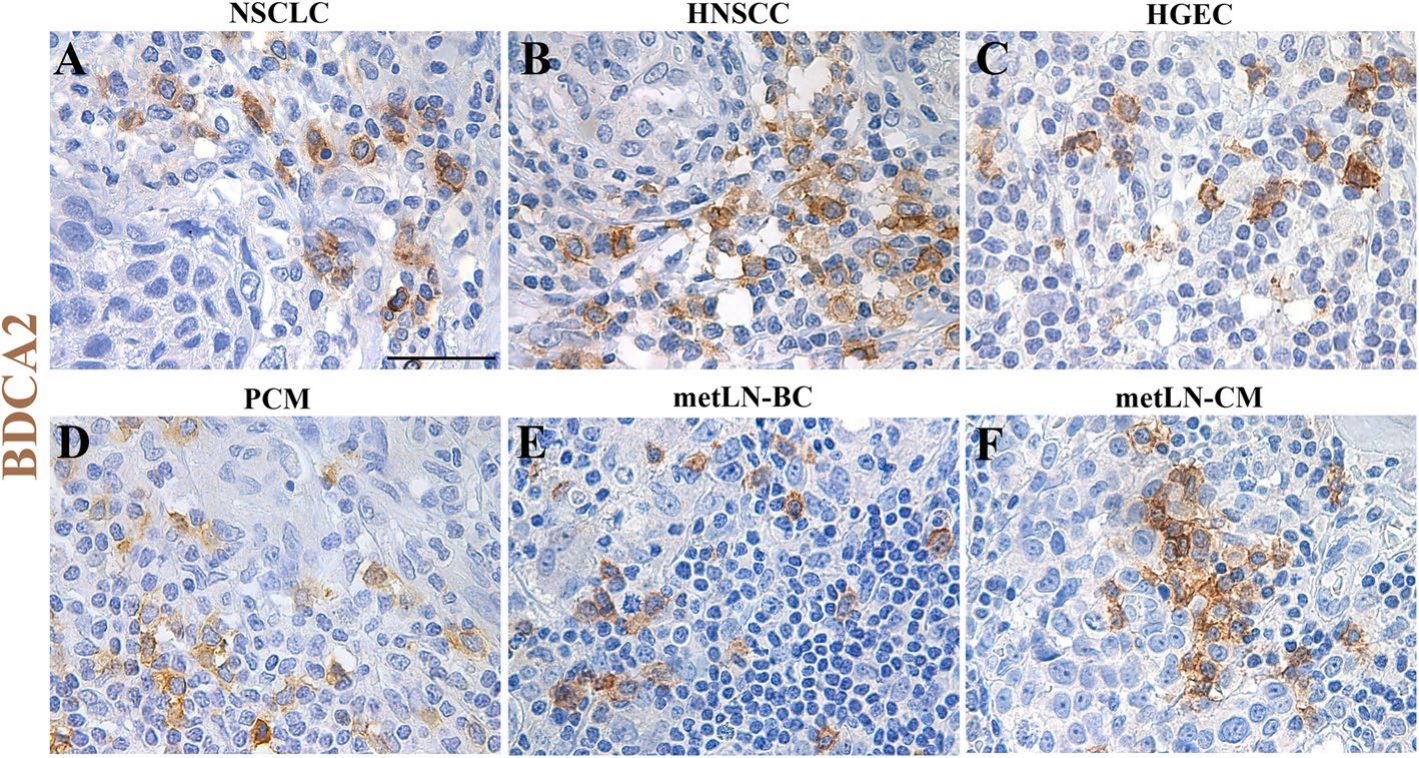
PDCs infiltrate primary tumors and nodal metastasis of various human cancer types. BDCA-2 staining on sections from primary [non-small cell lung cancer (NSCLC, **a**), head&neck squamous cell carcinoma (HNSCC, **b**), high grade endometrial carcinoma (HGEC, **c**), primary cutaneous melanoma (PCM), **d**] and nodal metastasis (metLN) [breast carcinoma (BC, **e**), cutaneous melanoma (CM, **f**)]. Magnification 400x; scale bar 50 µm

TA-pDCs have been detected at the primary tumor sites and in TDLNs of various cancer types (Fig. 5). Moreover, the density of TA-pDCs and the frequency of peripheral blood pDCs fluctuate among different human neoplasia and during disease progression (Table 1). As previously reviewed [3], TA-pDC accumulation was associated with a poor outcome in different cancer, such as BC [183, 197], ovarian carcinomas [182, 198], HNSCC [22] and OSCC [124, 199]. TA-pDCs were found in colon and colorectal carcinomas (CRC) [200–202], but they were associated with a different pathological stage and prognostic role [200, 201]. High numbers of pDCs were also found within HCC [203, 204] and this was correlated with greater vascular invasion, advanced N stage, higher recurrence rate, shorter OS and with FOXP3^+^ Tregs infiltration [203, 204]. In NSCLC [196, 205] and gastric cancer [206, 207], the elevated frequency of circulating and TA-pDCs was correlated with poor clinical outcome. Interestingly, pDCs infiltrating NSCLC facilitated tumor growth through the production of high levels of IL-1α in an AIM2-dependent manner [196]. By contrast, in lung adenocarcinoma (LUAD) patients with a smoking history, TA-pDCs were enriched and associated with favorable clinical outcomes [208]. The DNA-damaging anti-cancer treatments (i.e. ionizing radiation and DNA-alkylating agent), inducing the accumulation of tumor-derived DNA, might be involved in the recruitment of activated pDCs to the TME, highlighting the TLR9 upregulation by pDCs in mice treated with cisplatin or ionizing radiation [208]. Moreover, in a cohort of 288 patients with follicular lymphoma, the presence of pDCs was associated with increased OS [209]. PDCs were also present in the skin lesions of Kaposi's sarcoma (KS), associated with KS herpesvirus (KSHV, also known as HHV8) infection, but they were significantly less abundant as compared to inflamed Molluscum contagiosum cases [130]. pDCs were recruited to cervical carcinomas and OSCC after chronic HPV infection during the early steps of malignant transformation, but were absent before the infection, suggesting a potential role in antiviral response against HPV and favouring the clearance of pre-neoplastic lesions [107, 199, 210]. In CM, pDCs were found in the primary tumor lesions, whereas they were close to absent in nevi and normal skin [43, 99], and their high density predict a poor prognosis [186, 211]. The frequency of circulating pDCs was reduced in CM patients compared to healthy donors (HDs) [211], but their homing to sentinel lymph nodes was preserved [90, 99]. Moreover, our group demonstrated the collapse of tumor-infiltrating as well as circulating compartment of pDCs during melanoma progression [3, 99, 212]. Interestingly, an increase in pDC recruitment was observed in BRAFV600E melanomas compared to BRAF wild-type melanomas [99, 213]. In silico analysis of a pDC signature in pan-cancer TCGA datasets showed that a high pDC content was limited to TDLNs and stage I primary tumors, whereas pDC signature was poor in CM as compared to primary carcinomas [99, 126]. Instead, the pDC-specific signature has been significantly associated to a favorable survival outcome in the EBV-associated nasopharyngeal carcinoma, suggesting the potential role of pDCs in inducing antitumor immune responses [214]. Reduced frequency of circulating pDCs has been demonstrated in bladder cancer [215], CRC [216], pancreatic cancer [217], BC [218], and ovarian carcinoma [181] patients as compared to HDs and throughout disease progression. pDC frequency is reduced in acute lymphoblastic leukemia as well, which would be gradually restored after chemotherapy [219].

**Table 1:** The clinical significance of tumor-associated and circulating pDCs in human cancers

Tumor type	Patients’ sample (N°)	pDCs source	Detection method	pDCs frequency	pDCs functional state^a^	Clinical significance^b^	Ref
Ovarian cancer	Primary tumor (97)	Tissue (FFPE)	IHC (BDCA2^+^)	-	-	Negative (PFS)	[198]
Ovarian cancer	EOC (11)	Malignant ascites; Blood	FC (LIN^−^ CD123^++^ HLA-DR^+^)	Increased Pt ascites vs. Pt blood	-	-	[182]
Ovarian cancer	EOC (23)	Tissue (CF)	IHC (BDCA2^+^)	-	-	Negative (PFS)	[182]
Ovarian cancer	EOC stage I-IV (44)	Tissue (CS); Blood	FC (CD4^+^ CD123^+^ BDCA2^+^)	Decreased Pt tissue vs. Pt blood	Decreased	Negative (PFS)	[181]
HNSCC	Stage III-IV (16); TLN (16)	Tissue (CS)	FC (LIN^−^ CD123^+^ MHC-II^+^)	Increased Pt tissue vs. HD	Decreased	-	[22]
HNSCC	Primary tumor (45)	Blood	FC (LIN^−^ CD123^+^ HLA-DR^+^)	Unchanged	-	-	[308]
HNSCC	Tonsillar cancer lesions (24)	Tissue (CS)	FC (LIN^−^ CD123^+^)	Decreased Pt tissue vs. HD	-	-	[309]
OSCC	Primary tumor (6)	Blood	FC (CD123^+^ HLA-DR^+^)	Unchanged	Decreased	-	[199]
OSCC	Primary tumor (60)	Tissue (FFPE)	IHC (CD123^+^)	Increased Pt tissue vs. HD	-	Negative (OS, PFS)	[199]
OSCC	Primary tumor (10)	Tissue (CS)	FC (LIN^−^ CD123^+^ HLA-DR^+^)	Increased Pt tissue vs. HD	Decreased	-	[124]
OSCC	Primary tumor (10)	Tissue (FFPE)	IHC (CD123^+^)	Increased Pt tissue vs. HD	-	-	[124]
Breast cancer	Primary tumor (48)	Blood	FC (BDCA2^+^ CD123^+^)	Decreased Pt blood vs. HD	Unchanged	-	[183]
Breast cancer	Primary tumor (60)	Tissue (CS)	FC (BDCA2^+^ CD123^+^)	-	Decreased	-	[183]
Breast cancer	Primary tumor (151)	Tissue (FFPE)	IHC (BDCA2^+^)	-	-	Negative (OS, PFS)	[183]
Breast cancer	Primary tumor (255)	Tissue (FFPE)	IHC (CD123^+^)	Decreased Pt tissue vs. mDC	-	Negative (OS, PFS)	[197]
Breast cancer	Primary tumor (75)	Blood	FC (LIN^−^ CD123^+^ HLA-DR^+^)	Decreased in T II-III-IV vs. T 0-I	-	Positive (OS)	[218]
Colon cancer	Stage I-IV (149)	Tissue (FFPE)	IHC (BDCA2^+^)	Decreased during stages	-	Positive (OS, PFS)	[200]
Colon cancer	Primary tumor (58)	Tissue (FFPE)	IHC (BDCA2^+^)	Increased during stages	-	-	[201]
CRC	Primary tumor (63)	Tissue (FFPE)	IHC (CD123^+^)	Increased Pt tissue vs. HD	-	-	[202]
CRC	Primary tumor (26)	Blood	FC (CD123^+^ CD85k^+^)	Decreased Pt blood vs. HD	-	-	[216]
LUAD	Primary tumor (372)	Tissue (FFPE)	IHC (TLR9^+^ LILRA4^+^ IRF4^+^)	-	-	Positive (OS)	[208]
NSCLC	Primary tumor (14)	Tissue (CS)	FC (B220^+^ CD19^−^ BDCA2^+^ CD123^+^)	Increased Pt tissue vs. HD	-	-	[196]
NSCLC	Primary tumor (52)	Blood	FC (CD4^+^ CD123^+^ BDCA2^+^)	Increased Pt blood vs. HD; Increased in T III-IV vs. T I-II	-	-	[205]
HCC	Primary tumor (841)	Tissue (FFPE)	IHC (BDCA2^+^)	-	-	Negative (OS)	[203]
HCC	Primary tumor (117)	Blood; Tissue (CS); Malignant ascites	FC (LIN^−^ CD123^+^ MHC-II^+^)	Increased Pt blood; Tissue and ascites vs. HD	-	Negative (OS, PFS)	[204]
CM	SLN- (5); SLN + (5); mLN (5)	Tissue (FFPE)	IHC (CD123^+^)	Increased in mLN	-	-	[90]
CM	SLN- (19); SLN + (5); mLN (5)	Tissue (FF)	IF (BDCA2^+^)	-	Decreased	-	[90]
CM	SLN- (31); SLN + (8); mLN (9)	Tissue (CS)	FC (CD123^+^ BDCA2^+^)	Increased in SLN + /mLN vs. SLN-	-	-	[90]
CM	PCM (15); SLN- (2); SLN + (2)	Tissue (FF; FFPE)	IHC (CD123^+^ BDCA2^+^)	Increased in PCM vs. sk/nevi	Decreased	-	[43]
CM	PCM (397); SLN (71); MCM (25)	Tissue (FFPE)	IHC (CD123^+^)	Decreased in MCM vs. PCM; Increased in BRAF^V600E^ vs. BRAF^wt^	-	-	[213]
CM	PCM (12); mLN (28)	Tissue (CS)	FC (CD123^+^ BDCA2^+^)	Decreased in mLN vs. PCM	Functional	Negative (OS)	[186]
CM	Stage I-IV melanoma	Blood	FC (CD123^+^ BDCA2^+^)	Decreased in stage III-IV vs. I-II	Unchanged	-	[186]
CM	PCM (101); SLN (33); MCM (60)	Tissue (FFPE)	IHC (BDCA2^+^)	Increased in PCM vs. nevi; Increased in SLN vs. PCM; Unchanged in SLN + vs. SLN-; Decreased in MCM vs. PCM	-	-	[99]
CM	MM (29)	Blood	FC (CD123^+^ BDCA2^+^)	Decreased in MM blood vs. HD	-	-	[99]
CM	Stage I-IV Melanoma (17)	Blood	FC (LIN^−^ BDCA2^+^)	Decreased Pt blood vs. HD	Decreased	Positive (OS)	[211]
CM	MM (27)	Tissue (CS)	FC (LIN^−^ BDCA2^+^)	Increased Pt tissue vs. HD	Functional	-	[211]
CM	MM (29)	Blood	FC (CD123^+^ BDCA2^+^)	Decreased Pt blood vs. HD	Decreased	Positive (OS)	[212]
Bladder cancer	Primary tumor (13)	Blood	FC (LIN^−^ CD123^+^)	Decreased Pt blood vs. HD	-	-	[215]
Pancreatic cancer	Primary tumor (20)	Blood	FC (LIN^−^ CD123^+^ HLA-DR^+^)	Decreased Pt blood vs. HD	-	Positive (one year survival)	[217]
Gastric cancer	Primary tumor (32)	Blood	FC (LIN^−^ BDCA2^+^)	Increased Pt blood vs. HD; Increased in TNM III-IV vs. TNM I-II; Unchanged in Pt mLN + vs. Pt mLN-	-	-	[206]
Gastric cancer	Primary tumor (91)	Tissue (FFPE)	IHC (BDCA2^+^)	-	-	Negative (OS)	[207]
Gastric cancer	Primary tumor (41)	Blood	FC (LIN^−^ CD123^+^ HLA-DR^+^)	-	-	Negative (OS)	[207]
ALL	Primary tumor (45)	Blood	FC (LIN^−^ CD123^+^ HLA-DR^+^ CD11c^−^)	Decreased Pt blood vs. HD	-	-	[219]
Follicular lymphoma	Primary tumor (288)	Tissue (FFPE)	IHC (CD123^+^)	-	-	Positive (OS)	[209]

*Pt* Patients, *LUAD* Lung adenocarcinoma, *CM* Cutaneous Melanoma, *ALL* Acute lymphoblastic leukemia, *PCM* Primary Cutaneous Melanoma, *r-PCM* Regressing Primary Cutaneous Melanoma, *sk* Normal skin, *SLN*^*−*^ Negative Sentinel Lymph Nodes, *SLN*^+^ Positive Sentinel Lymph Nodes, *mLN* Metastatic Lymph Nodes, *TLN* Tumor Lymph Nodes, *MCM* Metastasis of Cutaneous Melanoma, *MM* Metastatic Melanoma, *HD* Healthy Donors, *FF* Frozen Fixed tissue, *FFPE* Formalin Fixed Paraffinn Embedded tissue, *CS* Cell suspension, *CF* Crio-fixed tissue, *IHC* Immunohistochemistry, *IF* Immunofluorescence, *FC* Flow cytometry, *EOC* Epithelial Ovarian Cancer, *HNSCC* Head and Neck Squamous Cell Carcinoma, *OSCC* Oral Squamous Cell Carcinoma, *CRC* Colorectal Carcinoma, *NSCLC* Non-Small Cell Lung Cancer, *HCC* Hepatocellular Carcinoma

^a^IFN-α production compared to control

^b^prognosis associated to high pDC content

In pan-cancer TCGA datasets, the signatures of pDC and I-IFNs were not correlated, suggesting that pDCs might be partly dysfunctional in I-IFNs production in the TME [126]. The above-mentioned studies are limited to immunohistochemistry or flow cytometry approach and lack functional data on the role of pDCs in the TME (Table 1). These limitations could explain the wide heterogeneity that has emerged in pDC frequency in the blood and tissues of cancer patients and its correlation with clinical outcome. These caveats could be overcome by using valuable tools for a spatial resolution of cancer tissues (i.e. multiplex imaging, spatial transcriptomics).

### Metabolism and pDCs immune escape

#### Cell metabolism control of innate immune functions of pDCs

In recent years, the subject of cell metabolism in the regulation of innate and adaptive immune cell response, defined as immunometabolism, has assumed increasing importance [220, 221]. The main metabolic pathways involved in pDC homeostasis and functions are glycolysis, oxidative phosphorylation (OXPHOS) and fatty acid oxidation (FAO).

It has recently been demonstrated that, upon TLR stimulation, DCs underwent metabolic reprogramming, which was critical for their activation (i.e. cytokines production) and innate immune functions [222, 223]. Particularly, TLR agonists (i.e. LPS, R848, CpG-B) induced a rapid increase in the glycolysis in DCs differentiated from bone marrow in the presence of the GM-CSF (GM-DCs) [224]. Interestingly, in GM-DCs, the glycolysis fueling was linked to CCR7 oligomerization to promote cellular motility and homing to draining lymph nodes [225]. This mechanism could also be applied to pDCs that rely on CCR7 expression to migrate in response to CCL19/CCL21 [82]. Moreover, rapid I-IFN production by pDCs demands high energy from glycolysis [153, 226]. An increase in hypoxia inducible factor alpha (HIF1α) expression has been observed together with enhanced glycolysis and IFN-α production [226, 227] in both activated primary human pDCs and GEN2.2 cell line. In fact, HIF1α is involved in the modulation of glycolysis by increasing the expression of the glucose transporter 1 (GLUT1) in Hep-2 human cells [228]; however, this mechanism has to be further investigated in pDCs. Moreover, the inhibition of glycolysis in pDCs reduced IFN-α production as well as the expression of HLA-DR and co-stimulatory molecules CD80 and CD86 [226]. These results underline the crucial nature of glycolysis for the antiviral function of pDCs (e.g. IFN-α production), but also for pDC-mediated T cell activation. Interestingly, the inhibition of FAO and mTOR pathway in pDCs resulted in a limited expression of CD40 and CD86 [229, 230], suggesting that appropriate cell metabolism is pivotal in the regulation of the mature state of pDCs. Finally, the autocrine/paracrine IFN-α feedback loop sustained FAO that, in turn, was able to support the OXPHOS in pDCs stimulated with CpG-A [230]. TLR7/8 agonists upregulated the OXPHOS and glutamine metabolism in pDCs, leading to a higher production of IFN-α and an increased T cell response [231]. In fact, the inhibition of both OXPHOS and glutamine metabolism prevented autophagy-dependent pDC activation [231]. The amino acid (AA) metabolism is important for sustaining mitochondrial respiration in DCs, as described in a recent review [232]. Moreover, several AA transporters were involved in the mTORC1 complex activation after TLR9 stimulation, resulting in I-IFN and chemokine production by pDCs [233].

Monocyte-derived DCs (moDCs) engage both TLRs and RLRs upon viral encounter, whereas pDCs use the RIG-I pathway only in the later stages of antiviral responses. Therefore, RIG-I-mediated I-IFNs response requires less energy as compared to TLR signaling and was supported by OXPHOS [227]. The inhibitors of mitochondrial electron transport chain also impaired IFN-α production by TLR7/9 activated pDCs, highlighting the importance of OXPHOS in the regulation of innate pDC functions [234]. In contrast with previous published data [226, 227], Hurley et al. did not find evidence of the central role of glycolysis in sustaining pDC functionality; however, glycolysis and FAO were both involved in IFN-α production by pDCs.

To date, the literature on pDC metabolism is still partially contradictory likely due to different culture methods for pDCs and the use of distinct TLR agonists or viral particles to stimulate them.

#### Oncometabolite and nutrient deprivation impair pDC functions

**Fig. 6:**
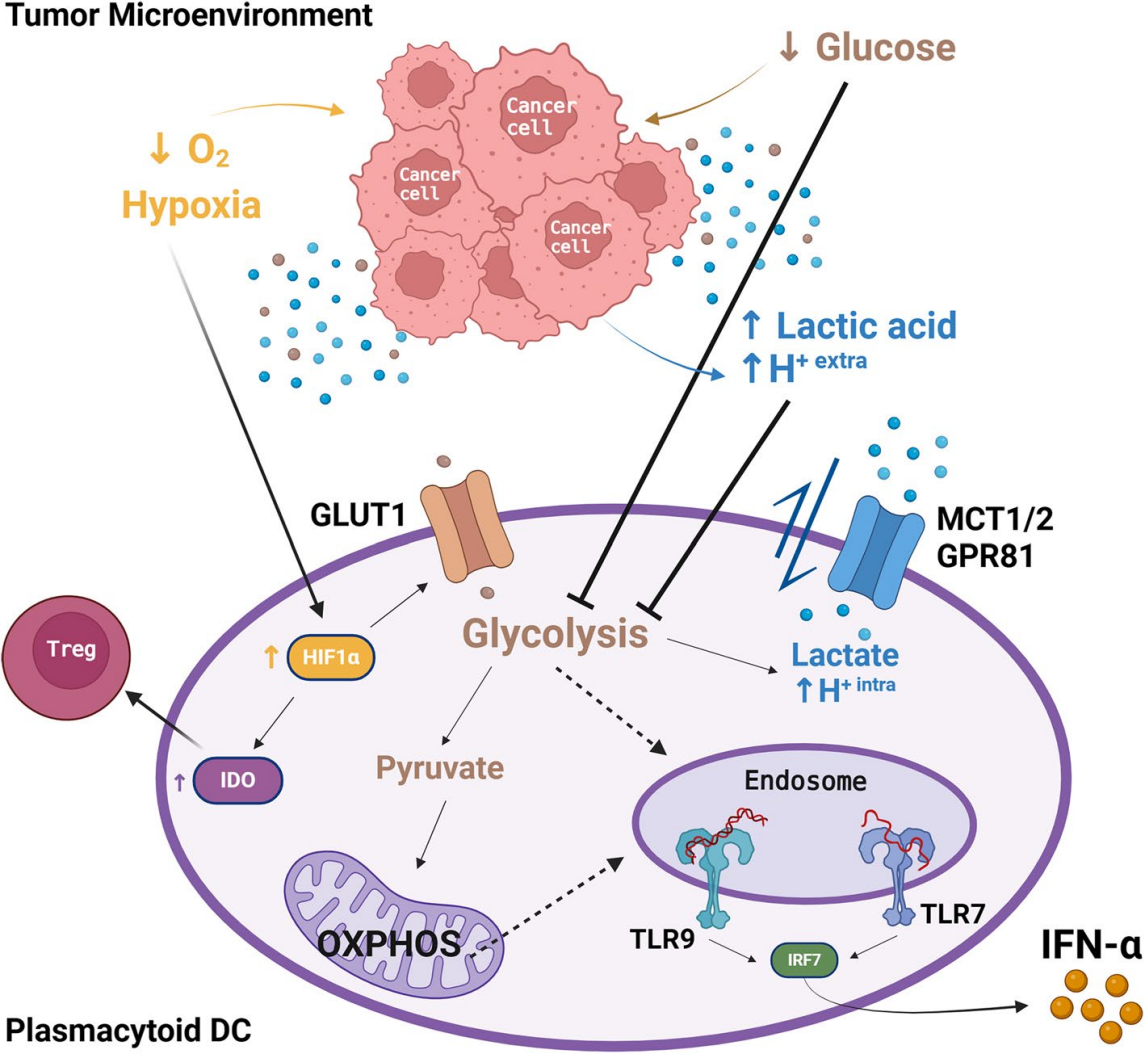
Tumor microenvironment negatively affects the cell metabolism and functions of human pDCs. Graphical illustration of the tumor microenvironment impact on pDCs metabolism. The IFN-α production by pDCs required high-energy demand from glycolysis. Cancer cells have a high glycolytic metabolism leading to both glucose deprivation (brown dots) and lactate accumulation (blue dots) with subsequent acidification of the extracellular milieu establishing an unfavourable environment directly hijacking the glycolytic capacity of pDCs. The inhibition of glycolysis could be also explained by the imbalance of import/export of lactic acid, mediated by the transporters MCT1/2 and the GPR81 receptor, leading to intracellular pH acidification. Moreover, oxygen deprivation results in HIF1α and GLUT1 increased expression on pDCs, supporting glycolysis rather than OXPHOS. Finally, the lactate accumulation and hypoxia modulate IDO expression by TA-pDCs and their tolerogenic switch. Solid lines indicate well-documented mechanisms; dashed lines indicate mechanisms based on limited evidences. Created with *BioRender.com*

Immunometabolism has revolutionized the tumor immunology landscape by unveiling how to manipulate metabolic pathways and promote antitumor immune responses [221]. Despite the increasing interest in immunometabolism as a novel strategy for cancer immunotherapy, the metabolic alterations of pDCs in cancer have been poorly investigated. Cancer cells have a high glycolytic activity that reduces glucose availability in the TME, with a subsequent lactate accumulation and acidification of the extracellular environment, known as the Warburg effect [152, 235]. Metabolic pathways are able to regulate the innate and adaptive immune responses to activation signals, including TLR agonists [236, 237] (Fig. 6). Hence, the in vitro and in vivo extracellular acidification can subvert pDC functionality, specifically by inhibiting their IFN-α production [238]. The inhibitory process is mediated by the interaction of lactic acid with lactate monocarboxylate transporters (MCT-1/2) and cell surface G-protein coupled receptor (GPR81) expressed on the pDC surface [238]. Lactate accumulation in the TME is responsible for the tolerogenic switch of TA-pDCs, particularly by eliciting IDO-dependent Treg generation [238]. Moreover, as described in the previous section, the hypoxic TME in HCC [190] and HNSCC [191] induced IDO upregulation by pDCs and thus their tolerogenic state. In particular, the pDC recruitment to the tumor site and upregulation of IDO were mediated by HIF-1α/eADO/ADORA1 signaling [190, 204]. All these data substantiated the idea that TME negatively affects pDC metabolism (i.e. glycolysis and OXPHOS) and their innate immune functions (Fig. 6).

Our group has previously demonstrated that CM secretome supported the collapse of circulating and TA-pDCs in advanced metastatic disease, with a potential role in lactic acidosis [99, 212]. We found that the lactate concentration was inversely associated to the concentration of glucose in melanoma-derived supernatants [212]. Mechanistically, in vitro exposure to melanoma soluble factors or lactic acidosis induced an IFN-α-defective tolerogenic state on fully differentiated pDCs [99, 126, 212]. Our recent data suggested that glucose deprivation and lactic acidosis by melanoma cells hindered glycolytic metabolism in pDCs, resulting ultimately in a detrimental effect on their IFN-α production [126, 212, 235] (Fig. 6). Accordingly, pDCs exposed to CM supernatants were characterized by metabolic drift and tolerogenic state [126]. Specifically, these data indicated a reduced ability of melanoma-conditioned pDCs to switch towards glycolysis to meet the cell energy demands [126]. In conclusion, the dysregulation of pDC metabolism in the TME could lead to defective antitumor function, including IFN-α production by pDCs.

### Modulating interferon production by pDCs: where do we stand?

Immunotherapies triggering an anticancer immune response remodel the tumor-infiltrating immune cells landscape. Immune checkpoint blockades (ICBs) have revolutionized the clinical management of various cancers. However, a consistent proportion of patients is refractory to ICB monotherapy and combination of double, or triple, ICB therapies lead to considerable side effects. Various immunostimulatory adjuvants, including agonists of PRRs (e.g. TLRs) or of their adapters (e.g. STING), have been developed to enhance the anti-cancer immunity induced by ICBs [239] and to overcome the resistance to systemic ICB therapies.

Preclinical studies have demonstrated that the recruitment of pDCs into neoplastic tissues and their activation represent a promising immunotherapeutic approach for various types of cancer [15, 154, 240–242]. Accordingly, therapeutic strategies to exploit the ability of pDCs to modulate tumor-specific T cell responses and cytotoxic functions have achieved promising antitumor effects in phase I/II clinical trials. To this end, two cancer immunotherapeutic strategies could be employed to enhance the immunostimulatory properties of pDCs: i) directly activating pDCs in vivo by targeting PRR signaling pathways; ii) exploiting autologous pDCs from peripheral blood or allogeneic pDC cell lines as cell therapy by developing novel pDC-based antitumor vaccines. Human pDC line derived from HLA-A*0201 leukemic pDCs (designated as pDC*line) showed the ability to initiate antitumor immune responses in humanized mice and in melanoma patients [243, 244].

Multiple clinical trials investigating the safety profile and therapeutic efficacy of PRR agonists, as monotherapy or in combination with chemotherapy, radiotherapy, or ICBs, have been started in the last decade (Table 2). However, only few clinical studies have been published at this time, but some of them have posted accessible results on *clinicaltrials.gov*.

**Table 2:** Clinical Trials investigating the TLR-7/9 agonists and STING agonists in human cancers

Molecule	Phase	Study (N°)	NCT Number
Imiquimod (IMQ)	I, Early	7	NCT04883645; NCT03276832; NCT03196180; NCT01678352; NCT03116659; NCT03276832; NCT04279535
	I	32	NCT03370406; NCT02689726; NCT01171469; NCT01264731; NCT00079300; NCT00142454; NCT02276300; NCT05055050; NCT01400672; NCT01902771; NCT03370406; NCT00788164; NCT00988559; NCT02234921; NCT01792505; NCT01795313; NCT06305910; NCT01808820; NCT01403285; NCT04808245; NCT04072900; NCT05375903; NCT00453050; NCT02600949; NCT01803152; NCT00944580; NCT03982004; NCT03872947; NCT01219348; NCT00118313; NCT05641545; NCT02454634
	I/II	8	NCT01421017; NCT02224599; NCT01421017; NCT00785122; NCT03559413; NCT01191034; NCT02452307; NCT02078648
	II	16	NCT03233412; NCT00899574; NCT02864147; NCT00031759; NCT01731652; NCT03180684; NCT00596336; NCT02802943; NCT00799110; NCT03534947; NCT00821964; NCT00273910; NCT00651703; NCT01909752; NCT01543464; NCT02293707
	II/III	4	NCT00941252; NCT02130323; NCT01088737; NCT00384124
	III	19	NCT02242929; NCT02059499; NCT02669459; NCT02329171; NCT01861535; NCT02669459; NCT01283763; NCT02394132; NCT00189280; NCT00189241; NCT00189306; NCT02059499; NCT00066872; NCT00129519; NCT01720407; NCT02242929; NCT05212246; NCT01212549; NCT02135419
	IV	6	NCT01161888; NCT00314756; NCT00204555; NCT01663558; NCT00803907; NCT00581425
	n.a	8	NCT02917746; NCT04859361; NCT03206138; NCT00504023; NCT00707174; NCT00463359; NCT00801320; NCT00685750
LHC165	I	1	NCT03301896
NJH395	I	1	NCT03696771
852A	I	2	NCT00095160; NCT00091689
	II	3	NCT00319748; NCT00276159; NCT00189332
SHR2150	I/II	1	NCT04588324
BDB001	I	2	NCT04196530; NCT03486301
	II	2	NCT03915678; NCT04819373
BDB018	I	1	NCT04840394
BDC-1001	I/II	1	NCT04278144
	II	1	NCT05954143
BNT411	I/II	1	NCT04101357
DSP-0509	I/II	1	NCT03416335
RO7119929	I	1	NCT04338685
TQ-A3334	I/II	1	NCT04273815
PF-3512676 (CPG 7909)	I	1	NCT00031278
	I/II	2	NCT00043420; NCT00185965
	II	7	NCT00321815; NCT00043368; NCT00880581; NCT00313768; NCT00471159; NCT00070642; NCT00070629
	III	2	NCT00254891; NCT00254904
SD-101	I	7	NCT02731742; NCT05607953; NCT03831295; NCT04935229; NCT01745354; NCT04050085;NCT0341090
	I/II	6	NCT05220722; NCT03322384; NCT02521870; NCT02927964; NCT02254772; NCT02266147
	II	1	NCT03007732
cavrotolimod (AST-008)	I/II	1	NCT03684785
CMP-001/ Vidutolimod	I	4	NCT03507699; NCT03438318; NCT03084640; NCT02680184
	I/II	3	NCT03983668; NCT04387071; NCT02554812
	II	8	NCT04708418; NCT04633278; NCT04698187; NCT03618641; NCT04401995; NCT04916002; NCT04807192; NCT05445609
	II/III	1	NCT04695977
	III	1	NCT05059522
IMO-2125/ Tilsotolimod	I	2	NCT04270864; NCT04196283
	II	3	NCT03865082; NCT04126876; NCT02644967
	III	1	NCT03445533
IMO-2055	I	3	NCT00633529; NCT00719199; NCT01360827
	II	2	NCT01040832; NCT00729053
TAC-001	I/II	1	NCT05399654
ADU-S100/ MIW815	I	2	NCT02675439; NCT03172936
	II	1	NCT03937141
MK-1454	I	1	NCT03010176
	II	1	NCT04220866
MK-2118	I	1	NCT03249792
E7766	I	2	NCT04144140; NCT04109092
BMS-986301	I	1	NCT03956680
GSK3745417	I	2	NCT03843359; NCT05424380
SB 11285	I	1	NCT04096638
BI-STING/BI 1387446	I	1	NCT04147234
TAK-676	I, early	1	NCT06062602
	I	1	NCT04879849
	I/II	1	NCT04420884
SNX281	I	1	NCT04609579
SYNB1891	I	1	NCT04167137
IMSA101	I/II	2	NCT04020185; NCT06026254
	II	2	NCT05846659; NCT05846646
DMXAA/ASA404	I	12	NCT00856336; NCT01290380; NCT00863733; NCT01299701; NCT01031212; NCT01278758; NCT00003697; NCT01299415; NCT01278849; NCT01240642; NCT01285453; NCT00674102
DMXAA/ASA404	I/II	1	NCT00832494
DMXAA/ASA404	II	3	NCT00111618; NCT01071928; NCT01057342
DMXAA/ASA404	III	2	NCT00738387; NCT00662597

*n.a.* Not applicable

#### Immunotherapy strategies based on TLR7/8 and TLR9 agonists

**Fig. 7:**
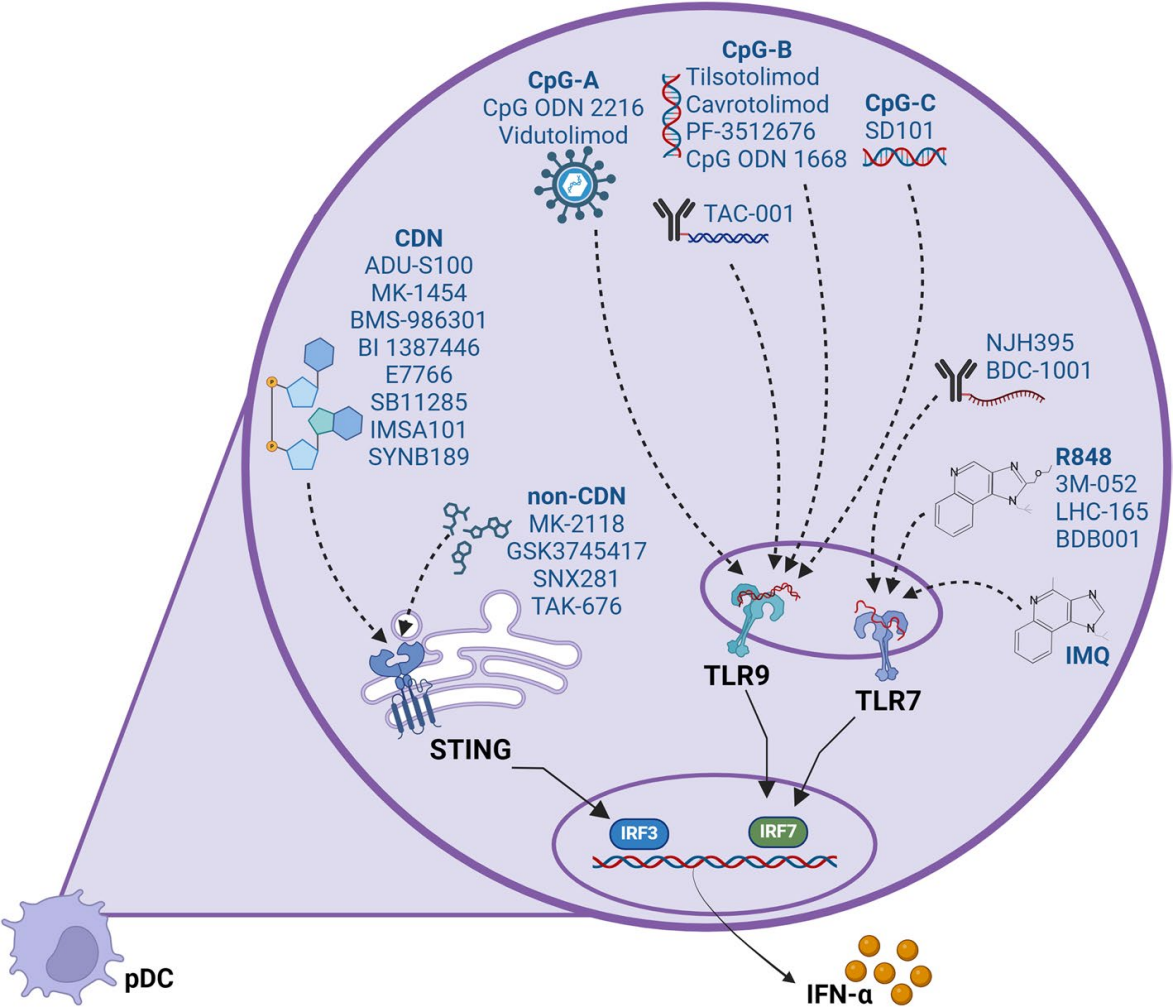
Synthetic TLR7/8, TLR9, and STING agonists in clinical trials. The immunostimulatory adjuvants can directly activate pDCs in vivo by targeting TLR or STING signaling pathways. Various TLR7/8, TLR9 and STING agonists are being used in multiple clinical trials, as monotherapy or in combination with ICB, to enhance anti-cancer immunity by exogenous activation of pDCs. IMQ is the first TLR7 agonist approved for the topical treatment of non-melanoma skin cancer. The TLR7/8 agonists include the second-generation (e.g. R848/resiquimod, BDB001, LHC165) and the third-generation (e.g. BDC-1001, NJH395) synthetic compounds. The unmethylated CpG oligodeoxynucleotides (CpG-ODN) are ligands of TLR9 and can be distinguished into three major classes: CpG-A (CpG ODN 2216, Vidutolimod/CMP-001), CpG-B (CpG ODN 1668, Tilsotolimod/IMO-2125, Cavrotolimod, CpG ODN 7909/PF-3512676, TAC-001), and CpG-C (SD101). Beyond natural cyclic dinucleotides (CDN), STING agonists include a variety of synthetic CDN (ADU-S100/ML RRS2 CDA, MK-1454, BMS-986301, BI 1387446, E7766, IMSA101, SB11285, SYNB189) and non-cyclic dinucleotides (non-CDN; MK-2118, GSK3745417, SNX281, TAK-676). Dashed lines indicate the potential binding of synthetic compounds to TLRs or STING. Solid lines indicate the well-known signaling pathways triggered upon TLR7/9 or STING activation. Created with *BioRender.com*

Preclinical data have demonstrated that administration of TLR7/8 and TLR9 agonists in combination with current anticancer regimens boosts the local and systemic antitumor immune response, promotes tumor cell killing and contributes to better outcomes, providing the rationale to use TLR agonists as adjuvants with ICBs [245–249]. Current efforts to use TLR agonists in clinical trials are largely focused on TLR7/8 and TLR9 agonists (Fig. 7), resulting in improved antitumor immunity mainly mediated by pDCs activation.

The Imiquimod (IMQ) was the first TLR7 agonist approved for the topical treatment of non-melanoma skin cancers (i.e. basal cell carcinoma) [250, 251]. The curative effects of IMQ have been linked to the activation of innate and adaptive antitumor immune mechanisms, in particular, by the recruitment of pDCs to the tumor site and trigger their cytotoxic functions [14, 162, 240]. These preclinical studies underlined the strong IMQ therapeutic potential. Specifically, the recruitment to and activation of pDCs in neoplastic tissues represent a promising immunotherapeutic approach for various types of cancer.

Several clinical trials have investigated topical IMQ in solid tumors (Table 2). Moreover, the combination of radiotherapy (RT) and IMQ generated an effective adaptive immune response, resulting in systemic control of metastatic breast cancer after local treatment of cutaneous metastases (NCT01421017).

Beyond IMQ, several synthetic TLR7/8 agonists showed promising immunostimulatory activity in preclinical models of solid cancers. These molecules have been further investigated for safety, tolerability, pharmacodynamics, pharmacokinetics, and efficacy profile as monotherapy or in combination with ICB, RT, or chemotherapeutics, in adults with advanced solid tumors (Table 2). However, none of these second- (e.g. R848) or third-generation (e.g. BDB001, BDC-1001, LHC165, NJH395) TLR7/8 agonists [252] (Fig. 7) has yet been approved by regulatory agencies for use in cancer patients. A novel injectable TLR7/8 dual agonist, 3 M-052, triggered innate immunity and activated systemic tumor-specific CD8^+^ T cell immunity, leading to the rejection of distant uninjected tumors without systemic cytokine release and toxicity [253]. However, pDCs and I-IFN were not indispensable for the antitumor activity of 3 M-052, although they are required for efficient tumor suppression [253]. NJH395 and BDC-1001 are novel immune-stimulator antibody conjugates (ISAC) combining an anti-HER2 antibody with TLR7/8 agonists aimed at delivering preferentially the immune activator into tumors and eliciting targeted immune modulation in the TME (i.e. IFN-I production), while lowering systemic exposure and minimizing immune-related toxicities [254], paving the way for combination treatment.

Among TLR9 agonists, unmethylated CpG oligodeoxynucleotides (CpG-ODN) can be divided into three major classes that differ in their molecular structure, endosomal trafficking, TLR9 downstream signaling, and pattern of immune cell activation: i) type A CpG-ODNs are mainly inducers of IFN-α production and activate pDC maturation, but weakly stimulate B cells; ii) type B CpG-ODNs strongly induce B-cell proliferation, cytokine production and have some effect on pDC maturation, but induce relatively scarce IFN-α secretion; iii) type C CpG-ODNs have intermediate immune stimulatory properties between type A and type B CpG-ODNs [255] (Fig. 7). Moreover, CpG-B and CpG-C possessed strong antitumor effects, but CpG-C was more rapid and effective than CpG-B in combination with the anti-PD-1 antibody [256].

Vidutolimod (formerly CMP-001) is a CpG-A ODN packaged within a virus-like particle composed of the bacteriophage coat protein Qβ [257]. Vidutolimod induced the production of anti-Qβ antibodies that formed immune complexes with virus-like particles, facilitating the uptake by pDCs expressing the costimulatory Fcγ receptor II [257]. Vidutolimod was specially designed to induce high levels of IFN-α, through activation of TLR9 in pDCs, which in turn induces a T helper 1 (Th1) response and has secondary effects on myeloid cells [128, 246, 257]. Additionally, in situ vaccination with CMP-001 triggered both local and abscopal antitumor immune responses [246, 249]. CMP-001 has been tested in combination with ICBs targeting CTLA-4 (i.e. ipilimumab), PD-1 (i.e. cemiplimab, nivolumab, or pembrolizumab) or PD-L1 (i.e. atezolizumab), alone or together with radiotherapy or surgery, in multiple neoplastic malignancies.

Tilsotolimod (also known as IMO-2125) is a type B CpG-ODN evaluated in co-treatment with ICB to test its efficacy in advanced solid tumors and melanoma [258]. Although, the ILLUMINATE-301 phase III study was terminated due to the lack of efficacy (NCT03445533). Interestingly, the administration CpG B-type ODN PF-3512676 (also known as CpG 7909) as immunostimulatory adjuvant therapy in stage I-III melanomas enhanced the activation of pDCs and CD1a^+^ mDCs, and lowered the frequency of immunosuppressive Treg, reducing the risk of metastatic spread in early-stage melanomas. In addition, the PF-3512676 administration was associated with increased I-IFN and proinflammatory cytokines, induction of activated CD86^+^ blood pDCs, and TRAIL^+^ IFN-induced monocyte-derived DC with a mature phenotype [259, 260].

Of relevance, SD-101 is a synthetic type C CpG-ODN able to stimulate human pDCs to release IFN-α, mature into efficient APCs and promote infiltration and expansion of CD8^+^ T cells expressing IFN-γ [261, 262], resulting in complete and long-lasting rejection of anti-PD-1 resistant tumors and effective systemic immunity against uninjected distant-site tumors [261, 262]. SD-101 has been studied in several clinical trials for the treatment of solid tumors, as monotherapy or in combination with pembrolizumab or IDO inhibitors alone or plus RT, or with anti-IL10 (Table 2).

Finally, the pDC cytotoxicity upon TLR7/8 or TLR9 agonists administration have been scarcely explored in clinical trials and future efforts should be designed accordingly.

The combination of ICB therapies with TLR7/8 and TLR9 agonists could turn “cold” tumors with an immunosuppressive microenvironment into immunologically “hot” tumors. Of note, preliminary studies suggest that TLR9 agonists might induce PD-1 surface expression on T cells and could enhance anti–PD-1 efficacy or reverse anti–PD-1 resistance [245–249] by enhancing systemic antitumor immune responses. For instance, PD-1 resistant melanoma patients could be eligible for this treatment [249]. Of relevance to this review, the IFN-α responsive genes in circulating leukocytes were measured as a surrogate of IFN-α production by TA-pDCs and were induced in all melanoma patients receiving i.t. SD-101 and pembrolizumab [248]. In responding patients, TA-pDCs were activated by vidutolimod to secrete IFN-α and induced Th1 antitumor immunity, including the rapid production of IFN-inducible chemokine CXCL10 and the generation of IFN-γ-secreting CD8^+^ T cells [249]. On the other hand, non-responding patients could have TA-pDCs resistant to TLR9 activation [5, 263], suggesting the need for early intervention during melanoma progression.

Critically, the expression of TLRs was also found on tumor cells. Particularly, TLR9 was strongly expressed on human tumor cell lines of different tissue-origin [264–266], including lung cancer [267], gastric carcinoma [268], cervical tumor [269], and prostate cancer [270], suggesting that TLR-signaling might modulate tumor development. However, the functional activity of TLR9 in tumor cells should be further investigated. The identification of the endogenous TLR ligands (i.e. nucleic acids released from dying cells) [271, 272] would clarify the mechanisms for tumor cell growth and potential off-target effects of TLR9 agonist administration. Moreover, one very recent report showed that NKp44^+^ pDCs physically interact with the platelet-derived growth factor D (PDGF-DD) expressing melanoma cells [273]. Therefore, PDGF-DD stimulation could enhance IFN-α secretion induced by the TLR9-mediated pDC response to self-DNA released by necrotic tumor cells. Finally, the expression of PDGF-DD by tumor cells should be taken into account in clinical trials using TLR9 agonists, such as CpG-ODN, as adjuvant therapy [273].

#### Controversial effect of STING agonists

In the TME, cancer cells are well-known to be repleted with cytosolic dsDNA derived from the rupture of micronuclear envelopes [274, 275], intrinsic DNA damage or exogenous genomic stress [275–278]. Tumor-derived DNA can activate the STING pathway at the level of cGAS in host immune cells or, alternatively, tumor-derived cGAMP can be transferred to host APCs thereby directly activating STING [279–281]. The activation of cGAS-STING pathway by tumor-derived dsDNA [241] has tremendous potential to improve antitumor immunity, playing an essential role in DC recognition of dying tumor cells, by generating I-IFN response and potently enhanced antitumor CTL responses [241, 242, 279, 282]. Although human pDCs could sense nucleic acids released from dying tumor cells [241, 279, 283], most TA-pDCs did not display endogenous activation. Notably, in melanoma, the endogenous activation of the cGAS-STING was limited to areas of spontaneous microscopic melanoma regression with a large number of tumor-infiltrating pDCs suggesting their activation [126].

Based on promising pre-clinical observations of host STING pathway involvement in endogenous local and systemic antitumor immune responses, the administration of STING agonists has been explored as a therapeutic strategy for the exogenous activation of pDCs resulting in prolonged survival and reduced tumor growth [242, 282, 284–286]. A variety of STING agonists, including natural or synthetic cyclic dinucleotides (CDN; e.g. c-di-GMP) and non-cyclic dinucleotides (non-CDN) with improved stability (Fig. 7), have been rapidly developed potentially to increase response rates to current immunotherapy approaches.

The flavonoid compound dimethyloxoxanthenyl acetic acid (DMXAA, also known as ASA404), a direct ligand for murine STING [287, 288], induced potent antitumor activity in mouse tumor models [242]. Several phase I-II trials have recently been initiated to investigate i.t. delivery of synthetic CDNs, such as ADU-S100, MK-1454, BMS-986301, BI 1387446, E7766, IMSA101, and SYNB189 (Table 2). Despite encouraging antitumor effects in preclinical models [242, 289], the phase I-II clinical trials of ADU-S100 (NCT03172936, NCT03937141, NCT02675439) and MK-1454 [290, 291] were terminated because no substantial antitumor activity was observed. The compounds BMS-986301, BI 1387446, and E7766 are currently in clinical testing in patients with advanced solid tumors that failed previous treatment, including ICB. Finally, non-cyclic dinucleotides (non-CDNs) include MK-2118, GSK3745417, SNX281, and TAK-676, that are being tested alone or in combination with pembrolizumab in patients with advanced solid tumors or lymphomas.

As previously described for TLR9 agonists, the activation of the cGAS-STING pathway is able to remodel immune-desert “cold” tumors into T cell-infiltrated “hot” tumors, by increasing antigen presentation, T cell trafficking and recruitment, and CTL functions. In addition, STING activation has been reported to induce upregulation of PD-L1. Therefore, combination therapies involving anti-PD-1/PD-L1 and STING are being evaluated in clinical trials (Table 2). However, the specific effects of STING agonists on the pDC-mediated antitumor response have been hitherto scarcely investigated.

In the oncology setting, it is also significant to consider the relevance of STING activation on cancer cells. The activation of cGAS-STING pathway in tumor cells can result in attenuated tumor growth, enhanced immunogenicity and susceptibility to lysis by tumor-infiltrating lymphocytes promoting tumor clearance. However, the selective pressure for tumor cells could decrease both cGAS and STING expression in human cancers protecting tumor cells [292]. Accordingly, defective STING expression has been reported in several melanoma or colorectal adenocarcinoma cell lines and in clinical cancer tissues, especially in advanced tumors [293–296]. On the other hand, emerging evidence suggested a pro-tumor role of the cGAS-STING pathway [297–299], which makes the clinical administration of STING agonists more challenging.

The combined immunotherapy of STING agonist with anti-PD-1/PD-L1 can neutralize the immunosuppressive effects of STING agonists (i.e. PD-L1 upregulation), delay tumor growth and protect against tumor rechallenge in mice [300, 301]. Based on this heterogeneity of outcomes, novel biomarkers for appropriate patient selection are mandatory to achieve a clinical response to this treatment.

#### pDC-based vaccines for cancer immunotherapy

Although the antigen-presentation capacity of conventional DCs (cDCs) is clearly defined, pDCs are generally attributed as having poor antigen-presentation function. The development of DC-based vaccines targeting tumor antigens that could be promptly cross-presented is a promising immunotherapeutic approach for cancer treatment. pDCs have the ability to present tumor antigens and prime tumor-specific cytotoxic CD8^+^ T cells [24, 25]. Human pDCs can expand antigen-specific CTLs in vitro. Depending on their activation state, pDCs can polarize proliferating T cells toward Th1, Th2, or Treg. However, we know very little about the antigen-presentation capacity of pDCs in vivo and their ability to elicit responses from T cells. Human pDCs are less efficient at presenting antigens as compared to cDCs, but whether they represent a distinct type of professional APC has yet to be clarified. Properly designed pDC-based immunotherapeutic approaches can boost tumor-specific immune responses and represent an attractive choice for treating cancer. Until now, most DC-based vaccination strategies are based on monocyte-derived DCs (moDCs) and cDC2s, with only a few clinical trials evaluating pDC-based vaccines. pDC-targeted vaccination elicited a strong cross-priming and durable CD8^+^ T cell response [27]. However, cross-presenting pDCs were unable to prime efficiently CD8^+^ T cells by themselves, requiring the cDC1s contribution [27]. Antigen transfer from pDCs to antigen-naïve cDCs for cross-priming was mediated by a unique mechanism of pDC-derived exosomes [27].

As previously reviewed [302], numerous clinical trials exploiting the antigen-presenting capacity of DCs have shown poor efficacy, probably due to immunosuppressive TME and the advanced disease stage of enrolled patients. A tolerogenic and dysfunctional immune phenotype in the TME could reduce the efficacy of DC-based vaccines.

Currently, peripheral blood autologous pDCs or allogeneic pDC cell lines derived from leukemic pDCs are tested for antitumor vaccine development [303, 304]. The first clinical trial that tested the therapeutic potential of pDC-based vaccine against malignant tumors was carried out in patients with MM expressing gp100 and tyrosinase (NCT01690377) [303]. Blood circulating pDCs were TLR-activated with FSME-IMMUN and loaded with melanoma-associated peptides before nodal injection. The activated pDCs displayed a mature phenotype and were able to migrate to lymph nodes. The upregulation of the IFN signature and tumor-specific T cell responses were also documented [303]. The vaccine was well tolerated, without evidence of severe toxicity, and improved PFS and OS in melanoma patients compared to controls receiving dacarbazine chemotherapy. A human pDC line derived from HLA-A*0201 leukemic pDCs was evaluated in a phase I clinical trial (NCT01863108) for the capacity to trigger antitumor responses in MM patients [304]. A similar approach was evaluated for lung cancer patients in a phase I/II trial (NCT03970746). A vaccine that combines the CD8^+^ T cell chemoattractive properties of pDCs with the superior tumor-antigen specific T cell priming capacity of cDC2s [305] was tested in patients with prostate cancer (NCT02692976), metastatic endometrial cancer (NCT04212377), castration-resistant prostate cancer (CRPC; NCT02692976), and metastatic endometrial cancer (NCT04212377). Functional antigen-specific T cells were detected in the peripheral blood of vaccinated patients and correlated with greater IFN-γ production and prolonged PFS [306]. Significantly, pDC-based cancer vaccines have been shown to increase the frequency of circulating antitumor T lymphocytes, together with the induction of I-IFN signature in patients with MM [303, 304, 307].

In some tumors, the administration of TLR agonists resulted in antitumor immune responses, combined with pDCs activation and clinical benefits suggesting that the development of pDC-based antitumor vaccines could represent an additional therapeutic option. The combination of pDC-based vaccination strategies with immune checkpoint inhibitors could further improve the clinical potential of pDCs [304].

## Conclusions

pDCs have been detected in a wide variety of human malignant neoplasms with different clinical outcome. Modulation of their effector and regulatory functions represent a novel window of intervention. However, intrinsic pDCs heterogeneity in term of immune functions should be highly considered. Moreover, TA-pDCs have been associated with a tolerogenic phenotype and functional impairment induced by immunosuppressive TME. Novel combinatorial immunomodulatory therapies targeting pDC activation can boost innate and adaptive cancer immunity.

## Data Availability

Not applicable.

## Abbreviations

2-DG: 2-Deoxyglucose
AML: Acute myeloid leukemia
APCs: Antigen presenting cells
BM: Bone marrow
BPDCN: Blastic plasmacytoid dendritic cell neoplasm
BST2: Bone marrow stromal cell antigen 2
cDC1s: Type 1 conventional dendritic cells
cDC2s: Type 2 conventional dendritic cells
CDN: Cyclic dinucleotides
CDP: Common dendritic cell progenitor
cGAMP: 2’,3’-Cyclic GMP-AMP
cGAS: Cyclic guanosine monophosphate-adenosine monophosphate synthase
CLP: Common lymphoid progenitor
CLRs: C-type lectin receptors
CM: Cutaneous melanoma
CMPs: Common myeloid progenitors
CpG-ODNs: CpG-rich oligodeoxynucleotides
CRC: Colorectal carcinomas
CTLA-4: Cytotoxic T-lymphocyte antigen 4
CTLs: Cytotoxic T lymphocytes
EBV: Epstein-Barr Virus
FA: Fatty acid
FAO: Fatty acid oxidation
Flt3L: Fms-like tyrosine kinase-3 ligand
GM-CSF: Granulocyte–macrophage colony stimulating factor
GPR81: G-protein coupled receptor
GrB: Granzyme B
HCC: Hepatocellular carcinoma
HD: Healthy donors
HEV: High endothelial venules
hGMDP: Human granulocyte-monocyte-DC progenitor
HHV8: Human herpes virus 8
HIF-1α: Hypoxia inducible factor alpha
hMDP: Human monocyte-dendritic progenitor
HNSCC: Head and neck squamous cell carcinoma
HPV: Human Papilloma Virus
HSPCs: Hematopoietic stem progenitor cells
ICB: Immune checkpoint blockade
ICOS-L: Inducible co-stimulator ligand
IDO: Indoleamine 2,3-dioxygenase
IFN-α: Interferon alpha
IFN-λ: Interferon lambda
I-IFN: Type I interferon
III-IFN: Type III interferon
IL-3: Interleukin-3
ILT7: Immunoglobulin-like transcript 7
IMQ: Imiquimod
IRF: Interferon Regulatory Factor
KSHV: Kaposi's sarcoma herpesvirus
LUAD: Lung adenocarcinoma
LYZ: Ysozyme
M-CSF: Macrophage colony stimulating factor
MLPs: Multipotent lymphoid early progenitors
MM: Metastatic melanoma
moDCs: Monocyte-derived dendritic cells
MPDCP: Mature PDC Proliferation
NK: Natural Killer cells
NLRs: NOD-like receptors
NSCLC: Non-small cell lung cancer
OS: Overall survival
OSCC: Oral squamous cell carcinoma
OX40-L/TNFSF4: Tumor necrosis factor ligand superfamily member 4
OXPHOS: Oxidative phosphorylation
PDC: Plasmacytoid Dendritic Cells
PDGF-DD: Platelet-derived growth factor D
PD-L1: Programmed death-ligand 1
PFS: Progression free survival
R848: Resiquimod
RLRs: RIG-I-like receptors
RT: Radiotherapy
STING: Stimulator of interferon genes
TA-pDCs: Tumor-associated pDCs
TDLN: Tumor-draining lymph node
TLR: Toll-like receptor
TME: Tumor microenvironment
TRAIL: TNF-related apoptosis inducing ligand
Th1: Type 1 helper T cells
Th2: Type 1 helper T cells
Treg: Regulatory T cell
